# Comparative transcriptome analysis reveals significant differences in the regulation of gene expression between hydrogen cyanide- and ethylene-treated *Arabidopsis thaliana*

**DOI:** 10.1186/s12870-019-1690-5

**Published:** 2019-03-04

**Authors:** Lulu Yu, Yang Liu, Fei Xu

**Affiliations:** 1 Applied Biotechnology Center, Wuhan University of Bioengineering, Wuhan, 430415 China; 2 College of Plant Science and Technology, Huazhong Agricultural University, Wuhan, 430070 China

**Keywords:** Hydrogen cyanide, Ethylene, *Arabidopsis thaliana*, RNA-seq

## Abstract

**Background:**

Hydrogen cyanide (HCN) is a small gaseous molecule that is predominantly produced as an equimolar co-product of ethylene (ET) biosynthesis in plants. The function of ET is of great concern and is well studied; however, the function of HCN is largely unknown. Similar to ET, HCN is a simple and diffusible molecule that has been shown to play a regulatory role in the control of some metabolic processes in plants. Nevertheless, it is still controversial whether HCN should be regarded as a signalling molecule, and the cross-talk between HCN and ET in gene expression regulation remains unclear. In this study, RNA sequencing (RNA-seq) was performed to compare the differentially expressed genes (DEGs) between HCN and ET in Arabidopsis. Gene Ontology (GO) and Kyoto Encyclopedia of Genes and Genomes (KEGG) analyses were subsequently performed to investigate the function and pathway enrichment of DEGs. Parts of key genes were confirmed by quantitative real-time PCR.

**Results:**

The results showed that at least 1305 genes and 918 genes were significantly induced by HCN and ET, respectively. Interestingly, a total of 474 genes (|log_2_ FC| ≥1) were co-regulated by HCN and ET. GO and KEGG analyses indicated that the co-regulated genes by HCN and ET were enriched in plant responses to stress and plant hormone signal transduction pathways, indicating that HCN may cooperate with ET and participate in plant growth and development and stress responses. However, a total of 831 genes were significantly induced by HCN but not by ET, indicating that in addition to ET, HCN is in essence a key signalling molecule in plants. Importantly, our data showed that the possible regulatory role of a relatively low concentration of HCN does not depend on ET feedback induction, although there are some common downstream components were observed.

**Conclusion:**

Our findings provide a valuable resource for further exploration and understanding of the molecular regulatory mechanisms of HCN in plants and provide novel insight into HCN cross-talk with ET and other hormones in the regulation of plant growth and plant responses to environmental stresses.

**Electronic supplementary material:**

The online version of this article (10.1186/s12870-019-1690-5) contains supplementary material, which is available to authorized users.

## Background

Hydrogen cyanide (HCN) is a natural metabolite in bacteria, fungi and plants that has received special attention from scientists since the beginning of the nineteenth century, due to its toxic effect on living organisms [1, 2]. Acute or chronic exposure to HCN can lead to intoxication, mild to severe illness, and in extreme cases even death in humans and animals because a certain dose of HCN inhibits the activity of metalloenzymes, principally cytochrome *c* oxidase, the final enzyme in the respiratory electron transport chain [3]. In plants, HCN is an equimolar by-product of ethylene (ET) biosynthesis via the ACC pathway [1]. In certain physiological states, such as fruit ripening and flower senescence, and in many environmental conditions, such as flooding and chilling, ET biosynthesis is greatly induced and at the same time, HCN accumulats rapidly [4]. In addition, HCN is released from cyanogenic lipids and cyanogenic glycosides during tissue disruption, infection, or cyanogenic plant food processing such as cassava roots (*Manihot esculenta*). There are more than 3000 species of higher plants including ferns, gymnosperms and angiosperms produce cyanogenic glucosides that are actively cleaved to produce HCN [5].

ET is certainly one of the most important plant hormones and is involved in numerous biological processes, such as root growth and stem elongation, fruit ripening, senescence, response to pathogens, response to gravity or submergence stress [6, 7]. In contrast to the roles of ET in plants, HCN is generally regarded as a phytotoxic agent and plays a protective effect in plants against predators such as herbivores [8, 9]. However, abundant information indicates that HCN, apart from being toxic, plays a regulatory (perhaps signalling) function in many physiological processes, e.g. seed germination, nitrate assimilation or in plant responses to some environmental stimuli [2, 10–12]. It was shown that exogenous 1 mM HCN treatment significantly promoted seed dormancy breakage and stimulated germination by inducing the production of reactive oxygen species (ROS) or feedback regulation of ET synthesis and signalling transduction [13–15]. Chivasa and Carr (1998) reported that potassium cyanide (KCN; 0.5 mM) pre-treatment could protect plants against tobacco mosaic virus (TMV) infection [16]. Additionally, the research of Seo et al. (2011) indicated that exogenous cyanide (0.5 or 1.0 mM) treatments contributed to the resistance of rice to blast fungus [11]. Our previous study demonstrated that a lower concentration of cyanide (20 μM KCN) could enhance cucumber seedlings against abiotic stress, such as drought stress and salinity stress [12].

Despite the well-described effects of HCN on germination and stress acclimation, its molecular regulatory mechanism remains largely unknown. It is possible that HCN plays a positive role in ET biosynthesis feedback regulation under conditions where rapid HCN accumulation occurs [17]. Some evidence proposed that the beneficial effect of HCN in seed germination might be due to feedback regulation of ET synthesis and signalling transduction [13], while other evidence hypothesized that HCN might be an important defence signal rather than ET in plant resistance to biotic stress [11].

HCN may play a dual role in plants, depending on its concentration [2]. HCN may be used in defense against herbivores at high toxic concentrations and may have a regulatory function at lower concentrations. Generally, most HCN produced in plants is detoxified quickly by the key enzyme *β*-cyanoalanine synthase (CAS) [18]. The remaining HCN at a lower level, probably at a non-toxic concentration, may act as a signalling molecule involved in the control of some metabolic processes in plants [2]. In Arabidopsis, it was shown that the CAS is encoded by a small gene family of three members, *CYS-C1* (At3g61440), *CYS-D1* (At3g04940), and *CYS-D2* (At5g28020) [19]. The most abundant CAS enzyme is CYS-C1, which is localized in the mitochondria and contributes most of the CAS activity in root and leaf tissue [20]. Interestingly, Garcia et al. (2010) mentioned that the accumulation of HCN within the mitochondrion in the Arabidopsis *cys-c1* mutant can act as an inhibitor of root hair development but is not toxic to the plant [21]. They also found that HCN accumulation in *cys-c1* mutant plants presented an increased susceptibility to the necrotrophic fungus *Botrytis cinerea* and an increased tolerance to the biotrophic *Pseudomonas syringae* pv *tomato* DC3000 bacterium and *Beet curly top virus*, suggesting that HCN might act by stimulating the salicylic acid (SA)-dependent signalling pathway of the plant immune system [22, 23]. However, how HCN participates in plant growth and stress responses is still largely unknown.

The aim of this study, therefore, is to uncover the regulatory role of HCN in Arabidopsis and compare the significant differences in gene expression regulation with ET. RNA sequencing (RNA-seq) methods were used to analyse the differentially expressed genes (DEGs) after treatment with HCN and ET, compared to the control. The data in this study provide a valuable resource for further exploration and understanding of the detailed molecular mechanisms of HCN in plant growth and response to environmental stress, and also provide novel insight into the cross-talk between HCN, ET and other hormones in Arabidopsis.

## Results

### Illumina sequencing and gene function annotation

In this study, a total of nine cDNA libraries from HCN-treated, ET-treated and control (CK) seedlings were prepared and subjected to Illumina deep sequencing, with each group created in triplicate. After removing the adaptors and low-quality sequences, the Illumina sequencing generated 467,217,912 sequence reads and 58.4 Gb of sequence data (Table 1). The good sequence and good ratio of each library was over 99%. In addition, the GC content of each library was approximately 45%, and CycleQ30% was greater than 90% for each library. Thus, the quality and accuracy of the sequencing data were sufficient for further analysis. All reads were aligned to the Arabidopsis genome and the results showed that most of the reads matched Arabidopsis genomic locations. All the unigenes matched previously described sequences with approximately 92% coverage.

**Table 1:** Summary statistics based on the RNA-seq data

Items	CK1	CK2	CK3	ET1	ET2	ET3	HCN1	HCN2	HCN3
Total Reads Count (#)	51,411,652	50,093,432	52,262,894	52,902,026	55,269,380	52,933,184	50,501,232	52,499,638	49,344,474
Total Bases Count (kp)	6,426,456.5	6,261,679	6,532,861.75	6,612,753.25	6,908,672.5	6,616,648	6,312,654	6,562,454.75	6,168,059.25
Average Read Length (bp)	125	125	125	125	125	125	125	125	125
Good sequences (#)	51,125,475	49,799,559	51,981,493	52,585,313	54,966,768	52,636,272	50,195,794	52,200,589	49,021,051
Good ratio (%)	99.44	99.41	99.46	99.4	99.45	99.44	99.4	99.43	99.34
Q30 Bases Ratio (%)	91.20	91.13	91.25	91.18	91.05	90.96	91.03	90.86	90.88
Q20 Bases Ratio (%)	95.72	95.67	95.76	95.70	95.65	95.60	95.62	95.55	95.53
GC Bases Ratio (%)	45.69	45.70	45.78	46.00	45.89	45.77	46.09	46.02	45.89
Total mapped	47,985,088 (94.31%)	46,340,714 (93.52%)	48,339,117 (93.42%)	48,925,996 (93.53%)	51,215,376 (93.62%)	49,011,714 (93.57%)	46,807,186 (93.74%)	49,092,621 (94.50%)	45,873,876 (94.11%)
Unique mapped	47,004,223 (92.38%)	45,813,306 (92.46%)	47,788,558 (92.36%)	48,310,664 (92.36%)	50,594,553 (92.49%)	48,390,954 (92.38%)	46,222,496 (92.57%)	48,488,187 (93.34%)	45,300,714 (92.93%)

**Fig. 1:**
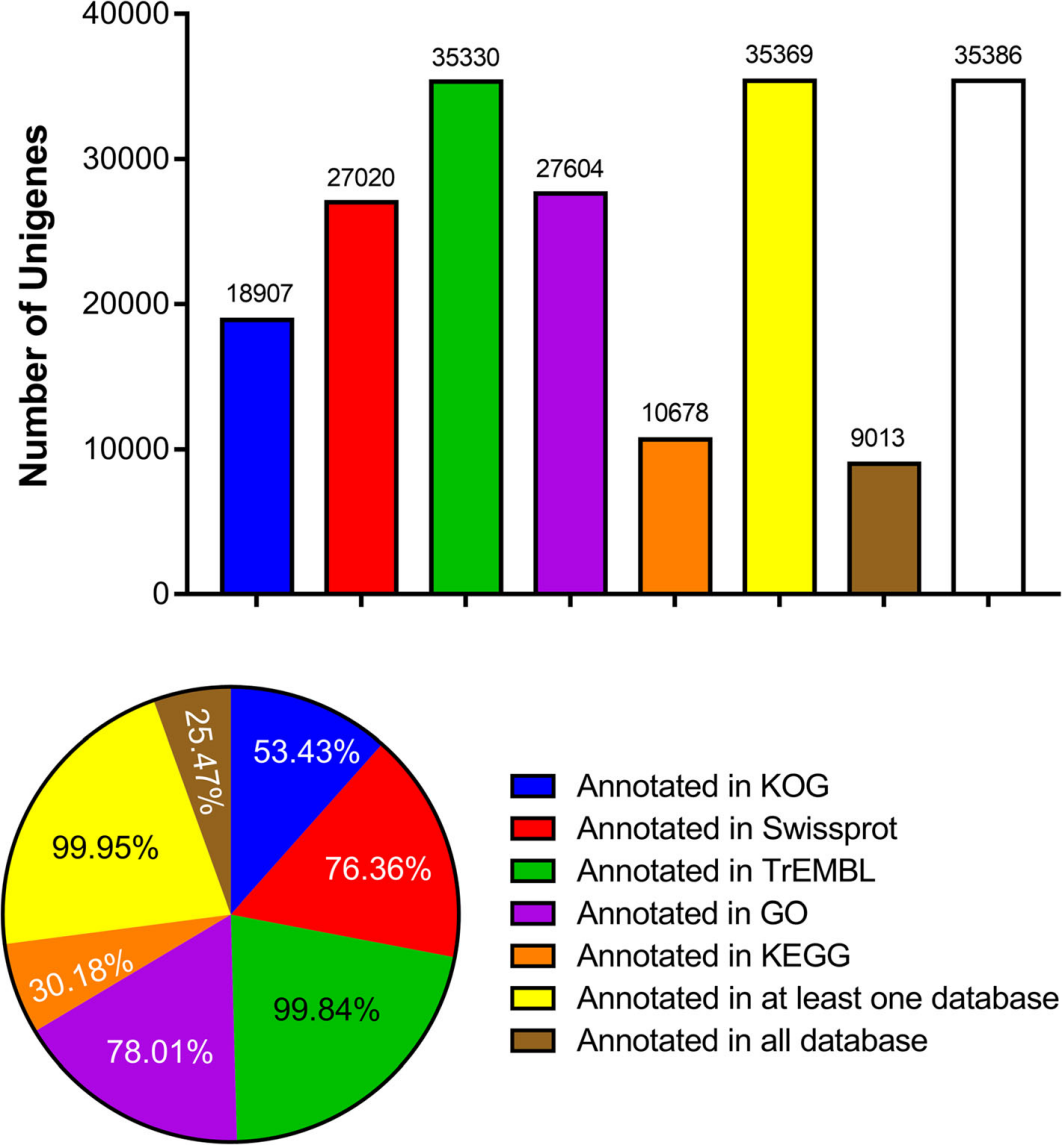
The number of Unigenes analyzed in this experiments

To elucidate potential gene functions, the gene annotation was carried out against KOG, SwissProt, TrEMBL, GO, KEGG databases. As shown in Fig. 1, there were 18,907, 27,020, 35,330, 27,604 and 10,678 unigenes were annotated in KOG, SwissProt, TrEMBL, GO, KEGG database, respectively. A total of 35,369 unigenes (99.95%) were successfully annotated in at least one database, and 9013 unigenes (25.47%) were successfully annotated in all databases (Fig. 1).

### Differentially expressed genes (DEGs) analysis

**Fig. 2:**
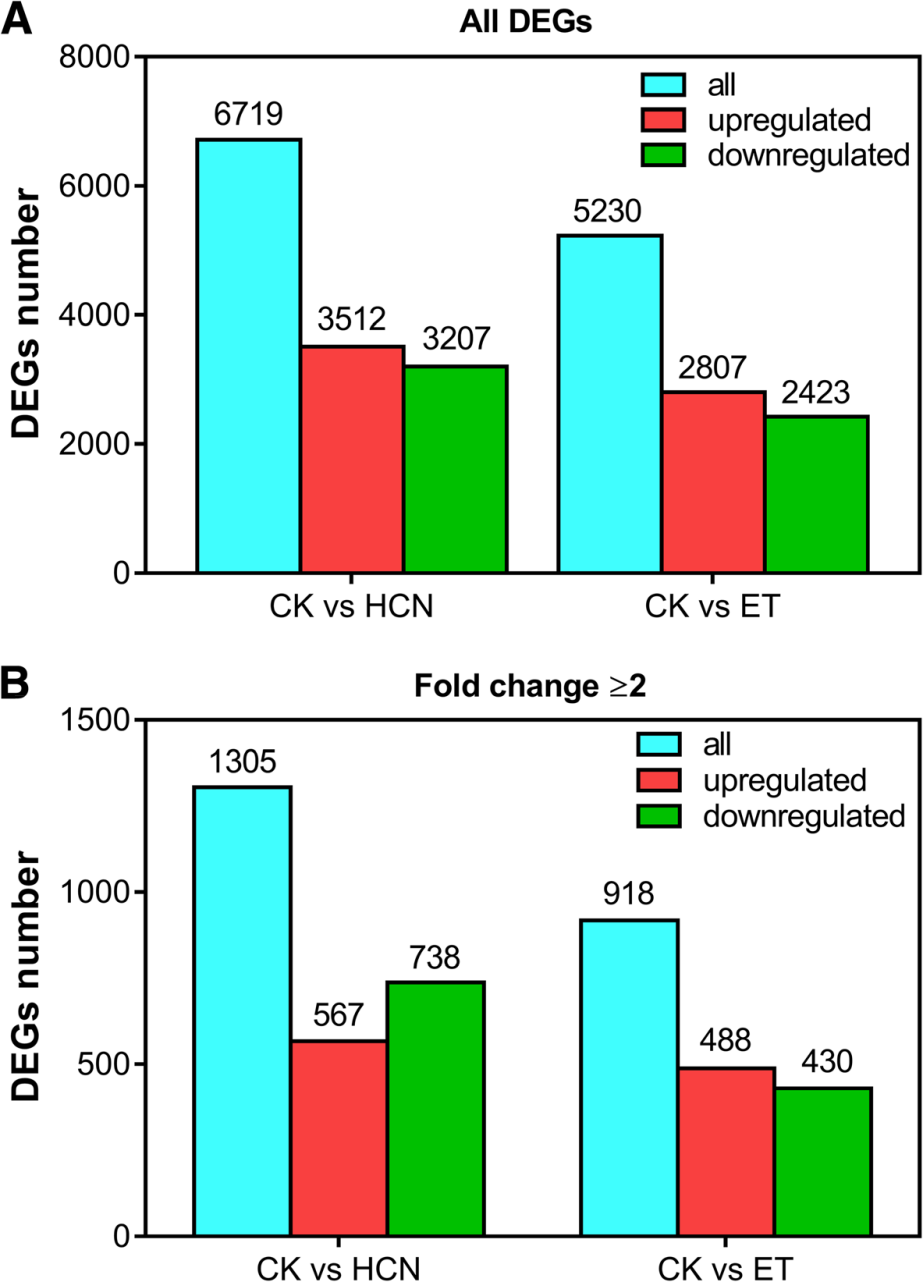
Comparison of DEGs number between CK vs HCN and CK vs ET. **a** Comparison of all DEGs regulated by HCN and ET. **b** The number of DEGs (fold change ≥2) regulated by HCN and ET was compared

To compare the upregulated or downregulated genes under the conditions of HCN and ET treatment, the differentially expressed genes (DEGs) were analysed according to the results of RNA-seq. As shown in Fig. 2a, a total of 6719 and 5230 DEGs were detected in the samples of CK vs HCN and CK vs ET, respectively. By comparison, a total of 3512 DEGs were upregulated and 3207 DEGs were downregulated in the HCN-treated samples (Fig. 2a), while 2807 DEGs upregulated and 2423 DEGs downregulated in ET-treated samples when compared with the control samples (Fig. 2a). If the absolute log_2_ fold change (log_2_ FC) ≥1 were used to judge the significance of differences in gene expression, a total of 1305 DEGs and 918 DEGs were detected in HCN-treated and ET-treated samples, respectively (Fig. 2b). There were 567 DEGs up-regulated and 738 DEGs down-regulated by HCN treatment, and 488 DEGs up-regulated and 430 DEGs down-regulated by ET treatment (Fig. 2b). These data indicated that although HCN is a co-product of ET, as expected it plays important roles in gene regulation in *Arabidopsis thaliana*.

To confirm the reliability of the transcriptome sequencing, parts of DEGs regulated by HCN or ET were investigated by qRT-PCR. As shown in Additional file 1: Figure S1, the results of qRT-PCR were generally consistent with the transcriptome data. In addition, linear regression analysis of the correlation between qRT-PCR and RNA-seq showed an R^2^ (goodness-of-fit) value of 0.9352 and a corresponding slope of 0.9322, suggesting a strong positive correlation between the qRT-PCR and transcriptome data.

### Genes commonly regulated by HCN and ET

**Fig. 3:**
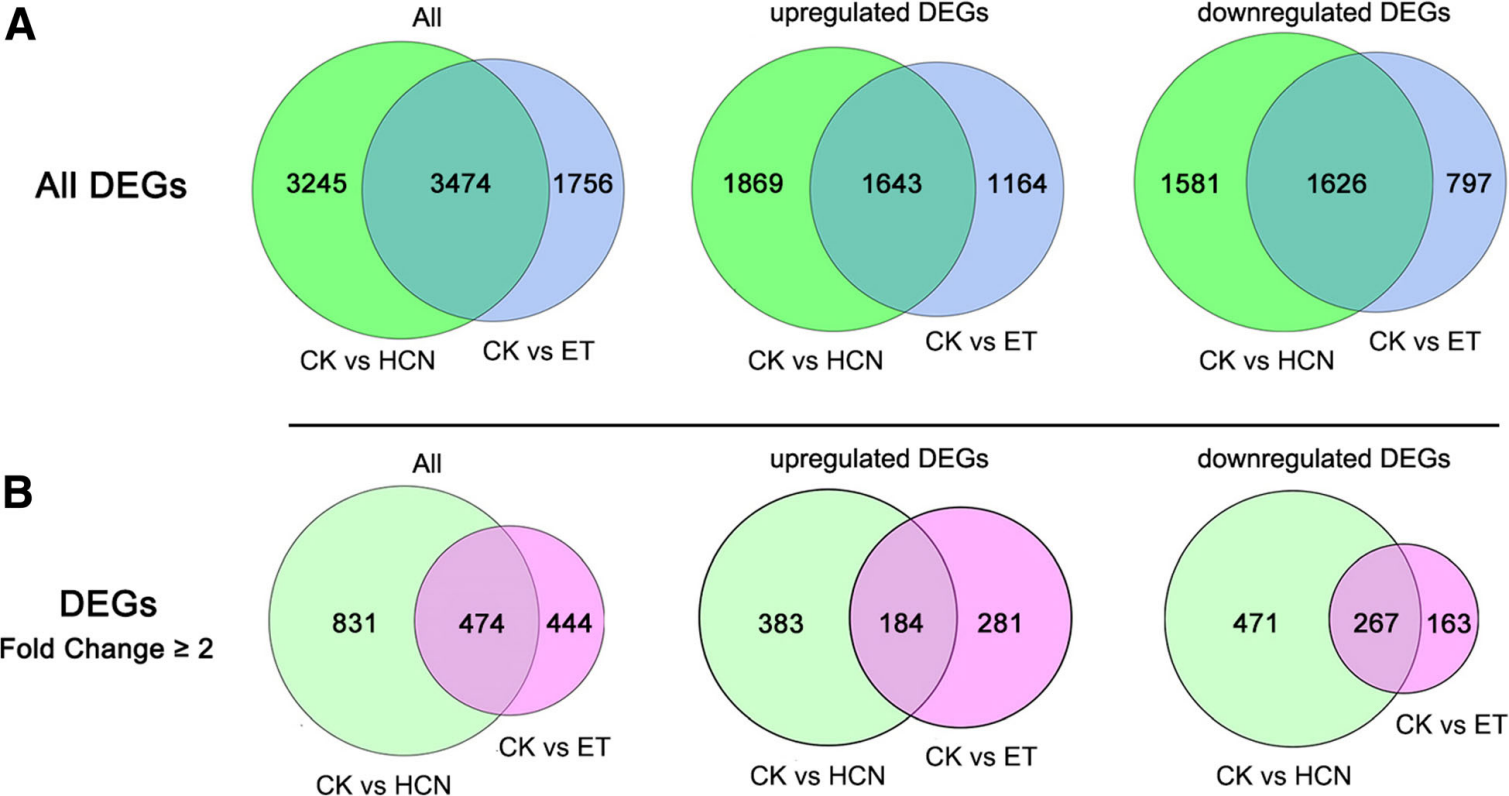
Venn diagram of DEGs regulated by HCN and ET. **a** Venn diagram of all DEGs from CK vs HCN and CK vs ET. **b** Venn diagram of DEGs (fold change ≥2) from CK vs HCN and CK vs ET

As mentioned above, HCN and ET may play regulatory roles in gene expression in Arabidopsis. Therefore, it is interesting to know how many genes were commonly regulated by both HCN and ET. The results showed that a total of 3474 DEGs were commonly regulated by HCN and ET, of which 1643 DEGs and 1626 DEGs were commonly upregulated and downregulated, respectively (Fig. 3a). As shown in Fig. 3b, a total of 474 genes were commonly regulated by HCN and ET when considering |log_2_ FC| ≥1. The comparison of DEGs between CK vs HCN and CK vs ET with a heatmap is shown in Additional file 1: Figure S2. Interestingly, among all of the 474 co-regulated genes (|log_2_ FC| ≥1), 184 genes upregulated and 267 genes downregulated by both HCN and ET, whereas only 23 genes were downregulated by HCN but upregulated by ET. The details of the top 10 commonly upregulated genes are listed in Table 2. Of these, the gene expression of *AT2G15020* was upregulated by 23-fold and 6-fold with treatment of HCN and ET, respectively, compared to the CK.

**Table 2:** Top 10 DEGs commonly upregulated by HCN and ET

Gene id	Gene annotation	CK vs HCN		CK vs ET	
		FC	Log_2_ FC	FC	Log_2_ FC
AT2G15020	Unknown protein	23.26	4.54	6.36	2.67
AT3G09440	HSP70/HSC70, HSP70 superfamily	12.64	3.66	9.00	3.17
AT4G03060	AOP2, encodes a 2-oxoglutarate- dependent dioxygenase	10.20	3.35	5.90	2.56
AT1G74310	Heat shock protein 101	8.28	3.05	4.89	2.29
AT2G46790	Pseudo-response regulator 9	8.00	3.00	2.73	1.45
AT3G14200	Molecular chaperone (DnaJ superfamily)	7.89	2.98	6.87	2.78
AT5G62730	H+/oligopeptide symporter	7.89	2.98	2.51	1.33
AT5G52640	Molecular chaperone (HSP90 family)	7.52	2.91	5.28	2.40
AT2G20560	Molecular chaperone (DnaJ superfamily)	7.46	2.90	5.74	2.52

*FC* fold change

### Genes exclusively induced by HCN or ET

**Fig. 4:**
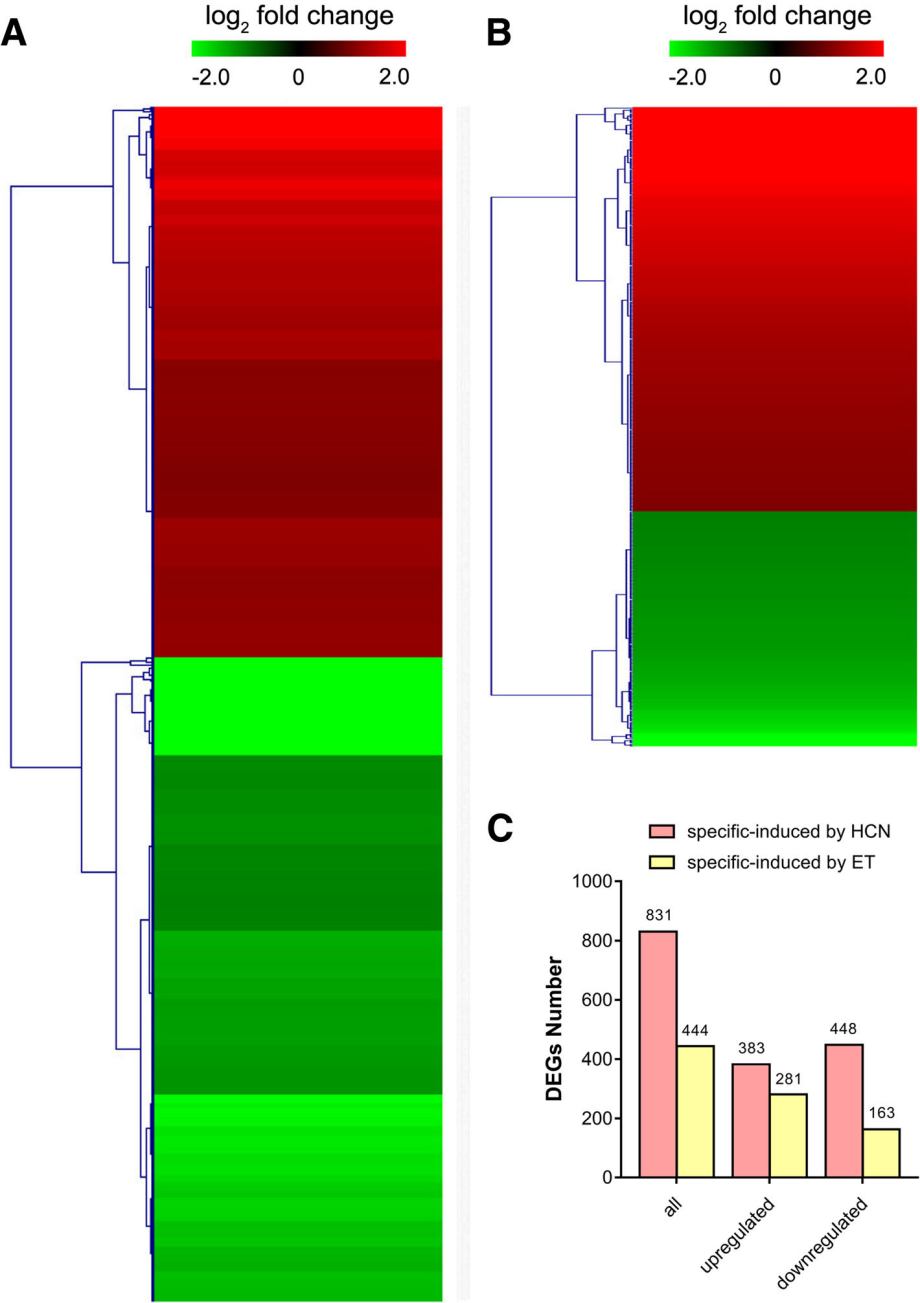
DEGs were exclusively-regulated by HCN or ET, respectively. **a** Heatmap of significantly regulated DEGs (fold change ≥2) by HCN. **b** Heatmap of significantly regulated DEGs (fold change ≥2) by ET. **c** The number of DEGs specific-regulated by HCN or ET, respectively

In addition to the co-regulated genes, the results showed that a total of 831 genes (|log_2_ FC| ≥1) were exclusively induced by HCN, including 383 genes that were upregulated and 448 genes that were downregulated by HCN (Fig. 4a). In comparison, there were approximately 444 genes (|log_2_ FC| ≥1) specially induced by ET, including 281 genes that were upregulated and 163 genes that were downregulated by ET (Fig. 4b). The details of the top 10 significantly upregulated genes by HCN or ET are listed in Table 3. It was shown that the DEGs significantly induced by HCN but not by ET included the genes *AT1G56600* (galactinol synthase 2, GolS2) and *AT5G37990* (S-adenosyl-L-methionine-dependent methyltransferase superfamily protein), which were 15-fold (log_2_ FC = 3.91) and 12.9-fold (log_2_ FC = 3.69) higher than in the CK. The DEGs significantly induced by ET but not by HCN included *AT3G11340* (UDP-Glycosyltransferase superfamily protein, UGT76B1) and *AT4G25490* (C-repeat/DRE binding factor 1, CBF1), which were approximately 12.8-fold (log_2_ FC = 3.7) and 9.6-fold (log_2_ FC = 3.3) higher than in the CK, respectively.

**Table 3:** Top 10 DEGs exclusively upregulated by HCN or ET

Samples	Gene id	Gene annotation	FC	Log_2_ FC
CK vs HCN	AT1G56600	GolS2, galactinol synthase 2	15.03	3.91
	AT5G37990	S-adenosyl-L-methionine-dependent methyltransferase superfamily protein	12.91	3.69
	AT1G70260	Nodulin MtN21 /EamA-like transporter family protein	10.85	3.44
	AT3G22840	Chlorophyll A-B binding family protein	10.48	3.39
	AT3G25190	Vacuolar iron transporter (VIT) family protein	6.82	2.77
	AT2G28780	P-hydroxybenzoic acid efflux pump subunit	6.63	2.73
	AT1G21340	Dof-type zinc finger DNA-binding family protein	6.32	2.66
	AT4G11320	Cysteine protease 2	5.86	2.55
	AT4G30250	P-loop containing nucleoside triphosphate hydrolases superfamily protein	5.78	2.53
	AT1G70850	MLP-like protein 34	5.06	2.34
CK vs ET	AT3G11340	UGT76B1, UDP-Glycosyltransferase superfamily protein	12.82	3.68
	AT4G25490	CBF1, C-repeat/DRE binding factor 1	9.65	3.27
	AT1G61255	Unknown protein	9.32	3.22
	AT4G22470	Protease inhibitor/seed storage/lipid transfer protein (LTP) family protein	9.00	3.17
	AT5G05340	PRX52, Peroxidase superfamily protein	8.11	3.02
	AT2G39518	Uncharacterized protein family (UPF0497)	8.11	3.02
	AT3G23250	MYB15, myb domain protein 15	7.78	2.96
	AT1G18290	DUF4228 domain protein	7.62	2.93
	AT1G05680	Uridine diphosphate glycosyltransferase 74E2	6.96	2.80
	AT4G24570	Dicarboxylate carrier 2	6.87	2.78

*FC* fold change

### GO and KEGG analysis show that the DEGs regulated by HCN are enriched in the plant hormone signal transduction pathway

**Fig. 5:**
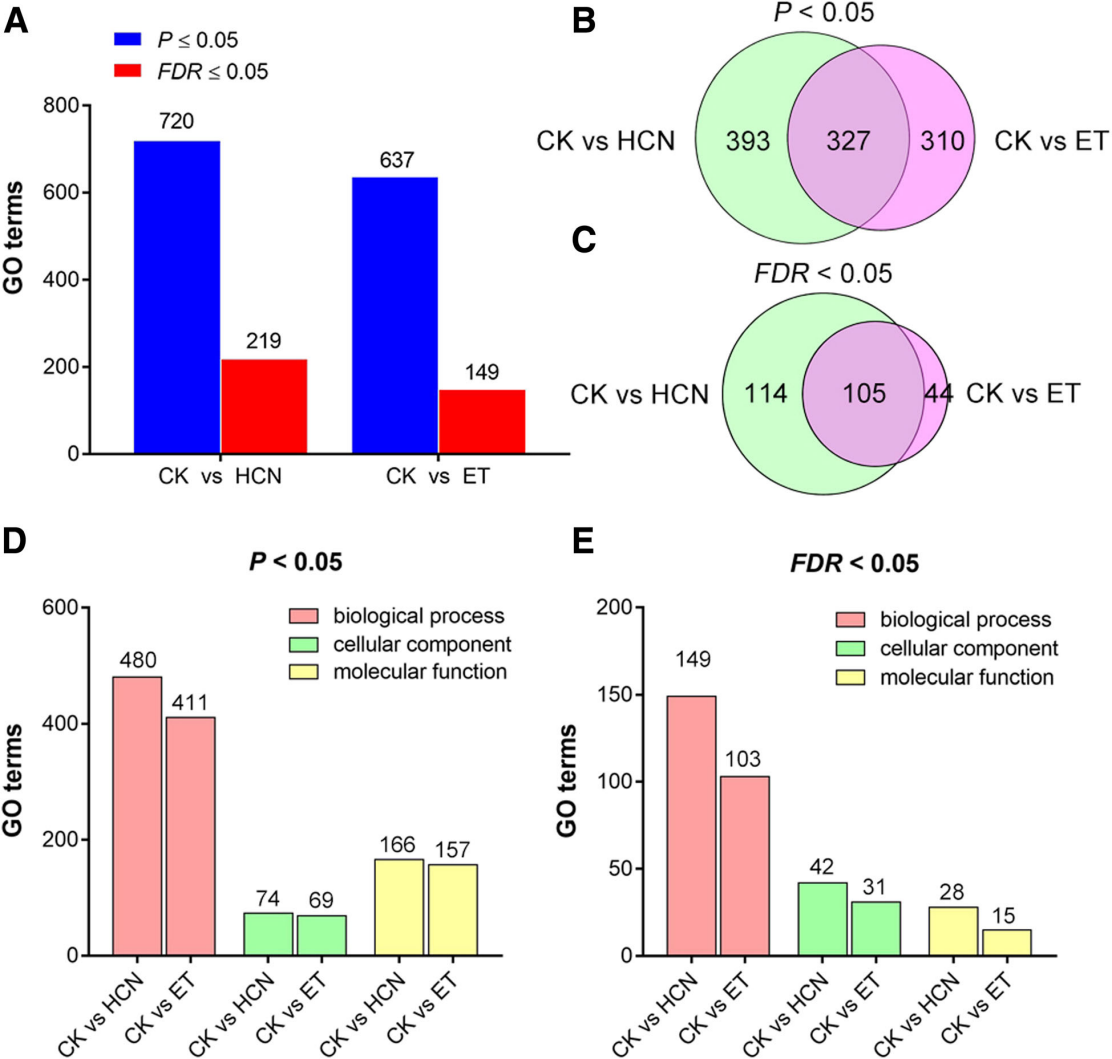
Comparison of GO terms enriched in CK vs HCN and CK vs ET. **a-c** Comparison of the numbers of GO terms between CK vs HCN and CK vs ET based on the *P*-values (*P* < 0.05) and false discovery rate (*FDR* < 0.05). **d** and **e** The GO categories of biological process, cellular component and molecular function were compared between CK vs HCN and CK vs ET based on the *P* < 0.05 and *FDR* < 0.05, respectively

**Fig. 6:**
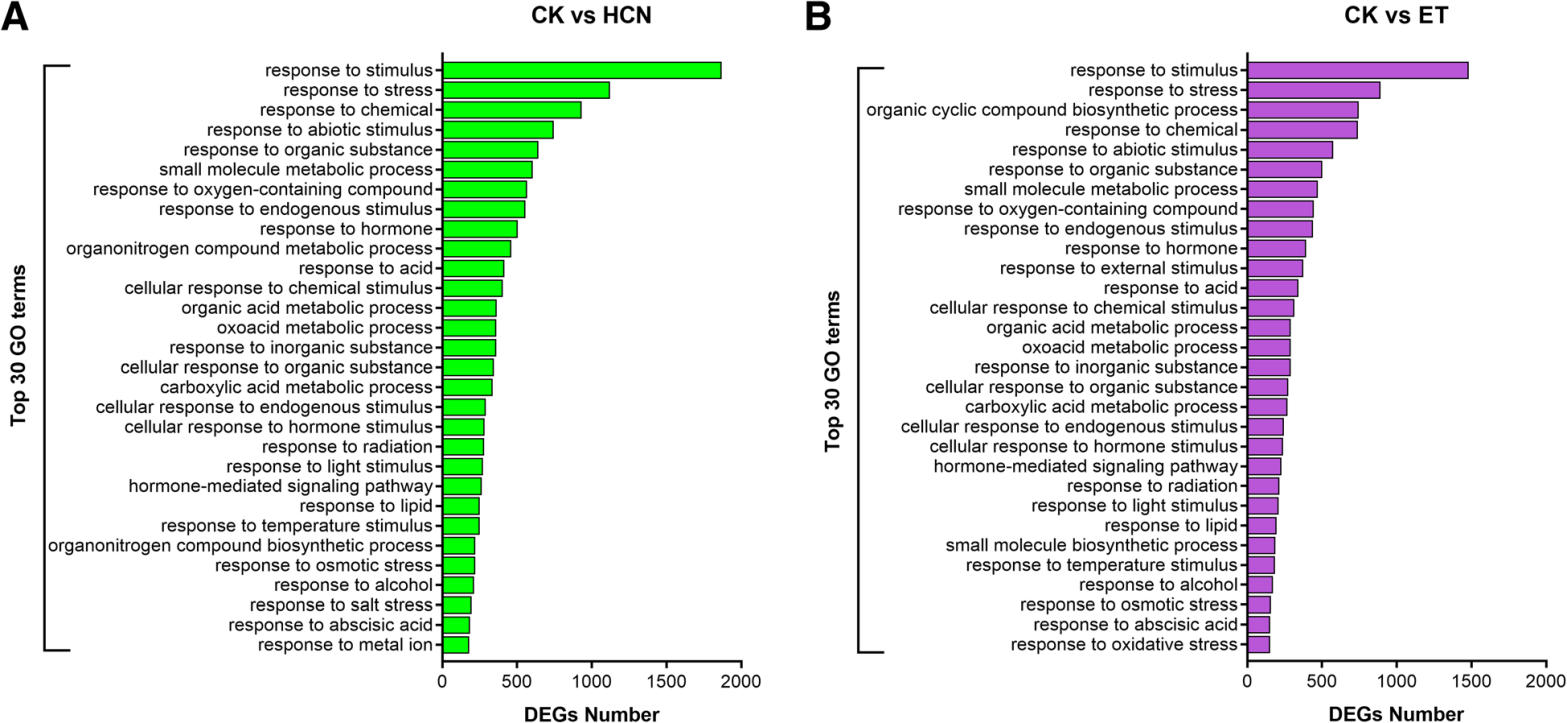
Comparison of top 30 GO terms between CK vs HCN and CK vs ET. The DEGs were assigned to biological process (BP) were analyzed and compared between them

To further investigate the functions of DEGs regulated by HCN or ET, GO and KEGG analyses were carried out. According to the GO analysis, the DEGs enriched in HCN-treated samples were assigned 720 GO terms (*P* < 0.05) and 219 GO terms (*FDR* < 0.05), while a total of 637 GO terms (*P* < 0.05) and 149 GO terms (*FDR* < 0.05) were significantly observed in ET-treated samples (Fig. 5a). There were 327 GO terms (*P* < 0.05) and 105 GO terms (*FDR* < 0.05) assigned to both HCN and ET (Fig. 5b, c). It should be noted that 481 GO terms of HCN regulated DEGs and 411 GO terms of ET regulated DEGs were associated with biological process (BP) (Fig. 5 d, e). In addition, there were no significant differences in the top 30 GO terms between HCN and ET, but the number of DEGs in each terms was significantly different between them (Fig. 6). The top 30 common GO terms assigned to BP between CK vs HCN and CK vs ET are shown in Additional file 1: Table S2, which shows that HCN- and ET-regulated DEGs were mainly enriched in plant responses to stimuli, environmental stress and hormones. These data further indicated that HCN and ET may be cooperatively involved in regulating plant growth and development, and plant resistance to environmental stresses.

**Fig. 7:**
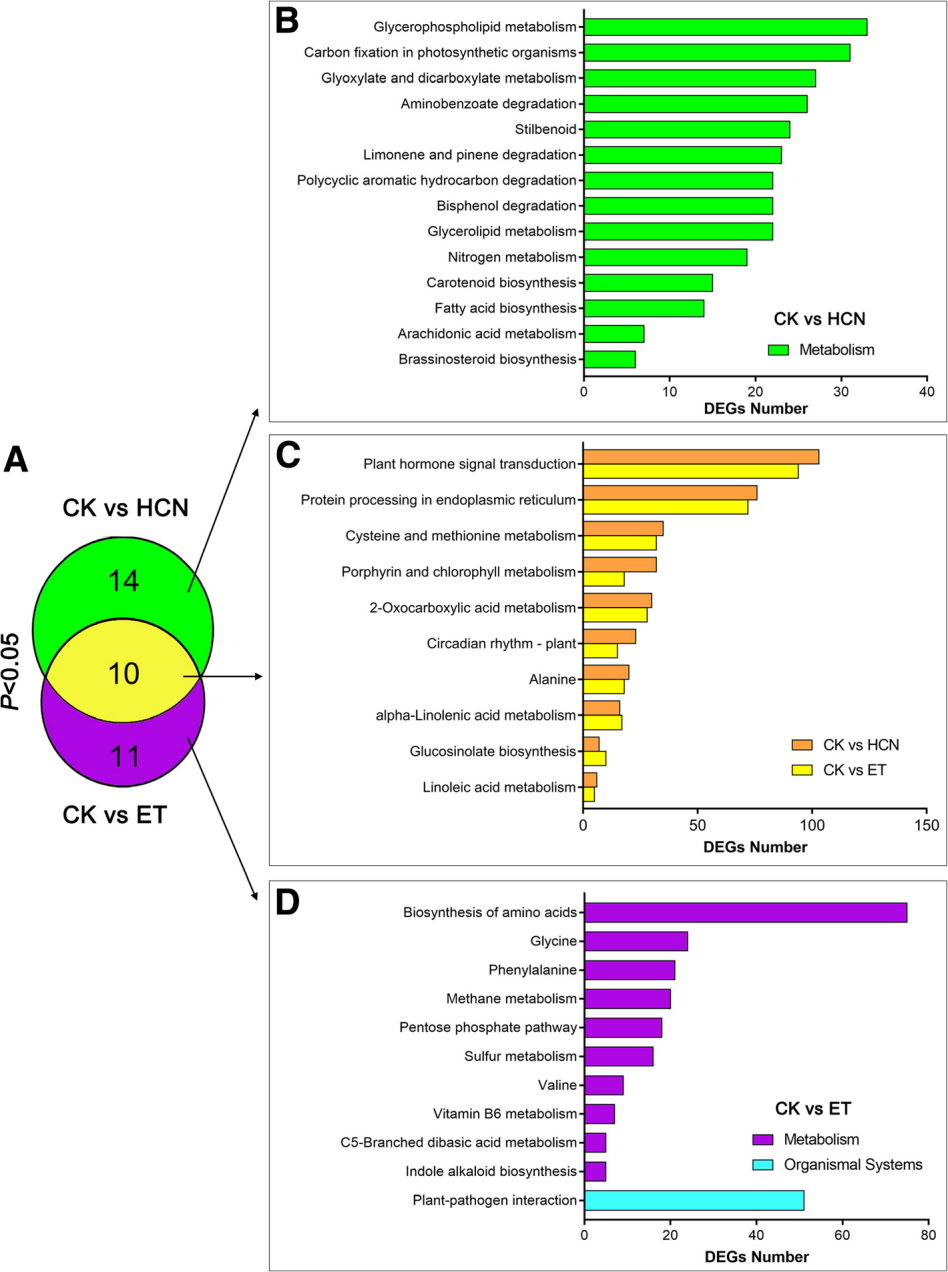
Comparison of KEGG pathways enriched in CK vs HCN and CK vs ET. **a** Comparison of KEGG terms (*P* < 0.05) between CK vs HCN and CK vs ET. **b** The KEGG pathways enriched in CK vs HCN. **c** The common KEGG pathways enriched in CK vs HCN and CK vs ET. (**d**) The KEGG pathways enriched in CK vs ET

KEGG analysis showed that the DEGs from CK vs HCN and CK vs ET were assigned to 24 and 21 KEGG pathways (*P* < 0.05), respectively (Fig. 7a). The common KEGG terms (*P* < 0.05) between CK vs HCN and CK vs ET were assigned to 10 pathways (Fig. 7a). Of these, the common pathway with the greatest number of DEGs was plant hormone signal transduction (ko04075) (Fig. 7c), consistent with the results of the GO analysis. Further analysis revealed that 103 DEGs (CK vs HCN) and 94 DEGs (CK vs ET) were assigned to the plant hormone signal transduction pathway (Fig. 7d). Among them, a total of 61 DEGs were co-regulated by both HCN and ET (Additional file 1: Figure S3 and Table S3). Interestingly, the genes co-regulated by HCN and ET were mainly associated with the auxin signalling transduction pathway, including the family genes from SMALL AUXIN UP RNAs (SAURs), GRETCHEN HAGEN3s (GH3s) and AUXIN/INDOLE ACETIC ACID (AUX/IAA) (Additional file 1: Table S3). Moreover, the common DEGs regulated by HCN and ET were also involved in the abscisic acid (ABA) signalling pathway (e.g., *HAB1*; hypersensitive to ABA1), cytokinin (CTK) signalling pathway (e.g., *ARR6*; cytokinin response regulator 6), gibberellin (GA) signalling pathway (e.g., *RGL2*; RGA-like 2) and brassinosteroid (BR) signaling pathway (e.g., *BSK2*; brassinosteroid signaling kinase 2) (Additional file 1: Table S3). These data further indicated that in addition to ET, HCN also engages in cross-talk with other plant hormone signalling molecules.

### The effect of HCN treatment on the gene expression of ethylene biosynthesis and signalling pathway

**Fig. 8:**
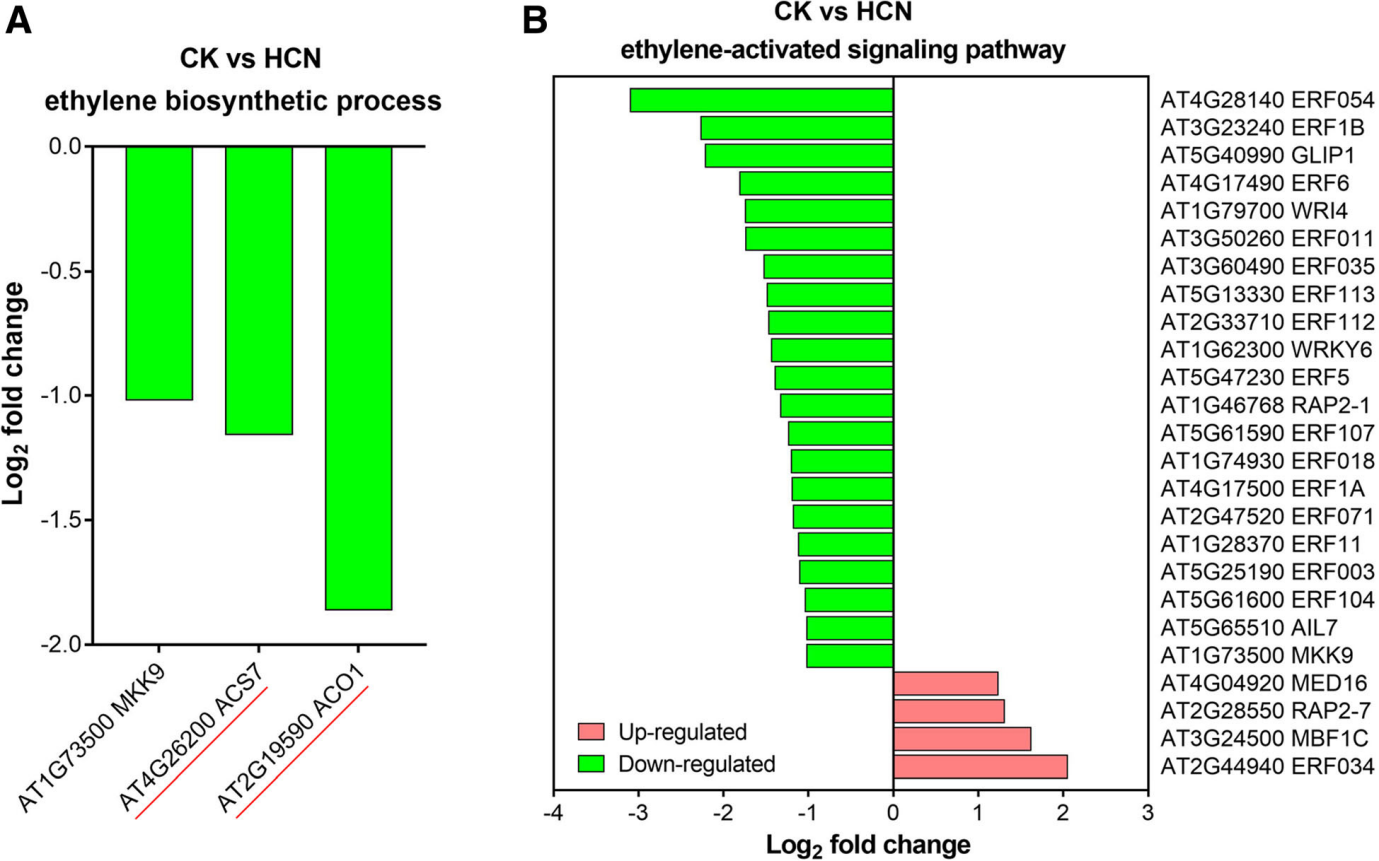
The DEGs involved in ethylene biosynthetic process and ethylene-activated signaling pathways from CK vs HCN. **a** The DEGs of CK vs HCN involved in ethylene biosynthetic process. **b** The DEGs of CK vs HCN involved in ethylene-activated signaling pathway

As mentioned previously, HCN is a co-product of ET synthesis and probably plays a role in plant growth and stress tolerance via the feedback-inducing synthesis of ET [13, 15, 17]. Therefore, the DEGs from CK vs HCN were analysed based on the GO annotation and KEGG pathway. As shown in Fig. 8a, there were no significantly up-regulated genes observed in the ET biosynthetic process (GO:0009693) with HCN treatment. In contrast, the gene expressions of 1-amino-cyclopropane-1-carboxylate synthase (e.g., *ACS7*) and 1-amino-cyclopropane-1-carboxylate oxidase (e.g., *ACO1*) were downregulated by HCN treatment, especially for the transcript of *ACO1*, a key gene for ET synthesis, which was downregulated more than 3.5-fold when compared to the CK. Further analysis of the ET-mediated signalling pathway (GO:0009873) showed that a total of 25 genes were significantly regulated by HCN, including 4 genes that were upregulated and 21 genes that were downregulated (Fig. 8b). For instance, the ET response factors including *ERF1B*, *ERF5* and *ERF6* were markedly down-regulated by HCN (Fig. 8b).

### The effect of HCN treatment on the gene expression of plant mitochondrial respiration

HCN poisoning is known to block the mitochondrial respiratory chain electron transport system by affecting complex IV [24]. Thus, the genes related to the mitochondrial respiratory chain (GO:0005746) were analysed according to the DEGs and GO enrichment analysis data. Interestingly, no significant DEGs (|log_2_ FC| ≥1) were found in HCN-treated samples compared to the CK (Table 4). Further analysis of all DEG isoforms (|log_2_ FC| < 1) showed that 3 genes were upregulated by HCN, namely, *AT5G25450* (cytochrome bd ubiquinol oxidase; fold change = 1.72), *AT3G10370* (FAD-dependent oxidoreductase family protein; fold change = 1.41) and *AT3G27240* (Cytochrome C1 family; fold change = 1.24) (Table 4). In contrast, the RNA-seq data showed that ET treatment significantly induced at least 11 genes (|log_2_ FC| ≥1) related to the mitochondrial respiratory chain, including cytochrome *c* oxidase and NADH dehydrogenase.

**Table 4:** DEGs related to mitochondrial respiratory chain pathway

Samples	Gene id	Gene annotation	FC	Log_2_ FC
CK vs HCN	AT5G25450	Cytochrome bd ubiquinol oxidase	1.72	0.78
	AT3G10370	FAD-dependent oxidoreductase family protein	1.41	0.50
	ATMG00730	Cytochrome *c* oxidase, subunit III	0.53	−0.92
	AT4G15010	Mitochondrial substrate carrier family protein	0.51	−0.96
	AT3G27240	Cytochrome C1 family	1.24	0.31
CK vs ET	AT2G07687	Cytochrome *c* oxidase, subunit III	4.92	2.30
	ATMG00650	NADH dehydrogenase subunit 4 L	4.38	2.13
	ATMG00160	Cytochrome *c* oxidase, subunit 2	4.26	2.09
	AT2G07689	NADH-Ubiquinone/plastoquinone (complex I) protein	4.08	2.03
	ATMG00730	Cytochrome *c* oxidase, subunit III	3.51	1.81
	ATMG01280	Cytochrome *c* oxidase, subunit 2 like	3.18	1.67
	ATMG00513	NADH-ubiquinone oxidoreductase chain 5	3.03	1.60
	ATMG00070	NADH dehydrogenase subunit 9	2.83	1.50
	ATMG00510	NADH dehydrogenase subunit 7	2.50	1.32
	ATMG00060	NADH-ubiquinone oxidoreductase chain 2, 5	2.46	1.30
	AT2G07727	Cytochrome b	2.31	1.21

*FC* fold change

In addition, it is noteworthy that HCN treatment induced, but not significantly, the gene expression of alternative oxidase 1A (*AOX1A*; fold change = 1.58) (Table 5), which is the HCN-insensitive protein that mediates HCN-resistant respiration. In contrast, the RNA-seq data showed that *AOX1D* (fold change = 0.17) was significantly downregulated by HCN. Moreover, HCN induced, but not significantly, the gene expression of cysteine synthase D2 (*CYSD2*; fold change = 1.38), which is involved in HCN detoxification. Importantly, the expression of genes involved in ROS production was not significantly affected by HCN treatment (Additional file 1: Table S5). In comparison, ET treatment induced, but not significantly, the expressions of *AOX1A* (fold change = 1.97) and *AOX1C* (fold change = 1.73) but slightly reduced the expression of *CYSD1* (fold change = 0.78).

**Table 5:** DEGs related to cyanide-resistant and cyanide degradation pathway

Samples	Gene id	Gene annotation	FC	log_2_ FC
CK vs HCN	AT3G22370	AOX1A, alternative oxidase 1A	1.59	0.67
	AT5G28020	CYSD2, cysteine synthase D2	1.38	0.46
	AT1G32350	AOX1D, alternative oxidase 1D	0.17	−2.56
CK vs ET	AT3G22370	AOX1A, alternative oxidase 1A	1.97	0.98
	AT3G27620	AOX1C, alternative oxidase 1C	1.73	0.79
	AT3G04940	CYSD1, cysteine synthase D1	0.80	−0.33

*FC* fold change

### Comparative analysis of stress-related genes between HCN and ET treatment

**Fig. 9:**
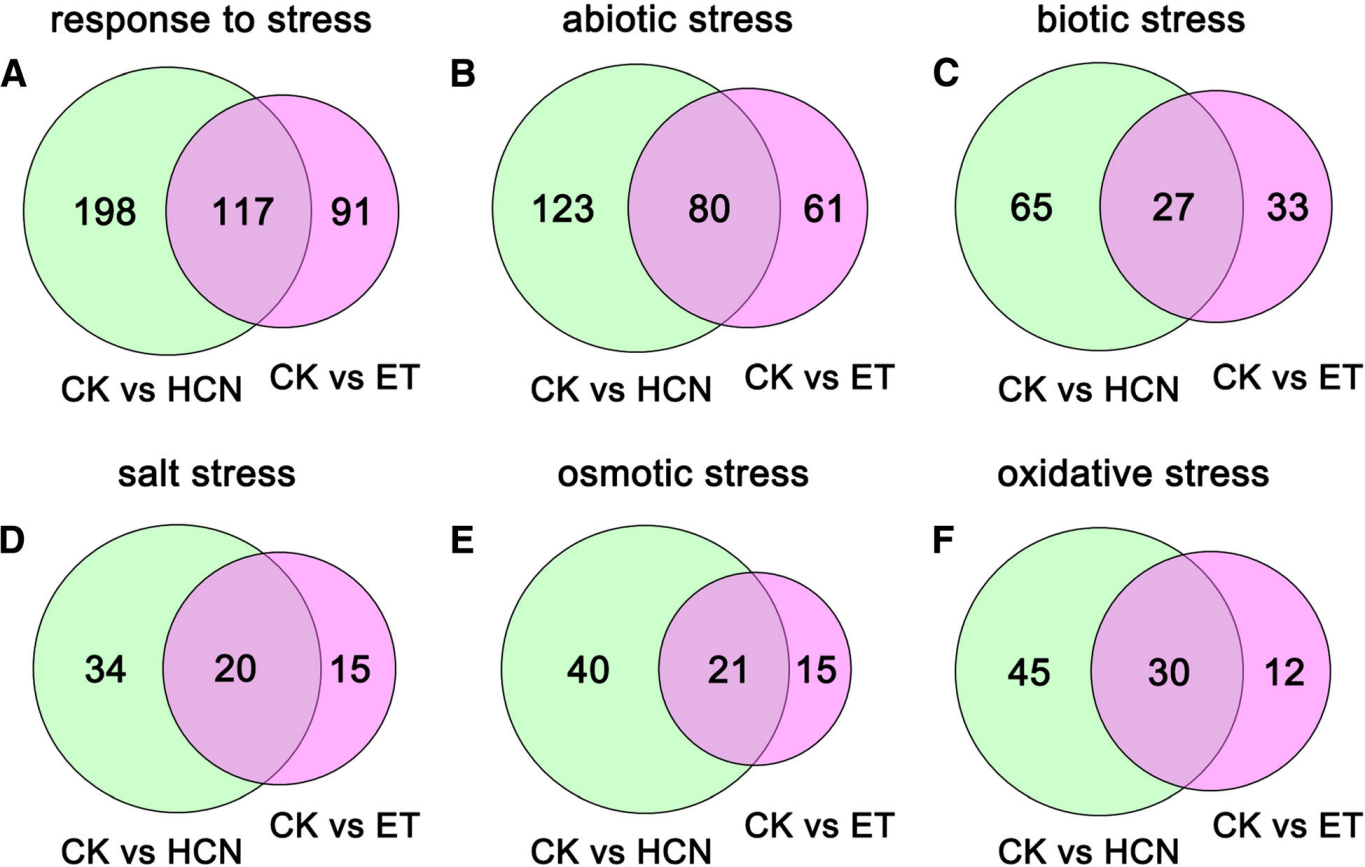
Comparison of stress-related genes that were regulated by HCN and ET. **a** Response to stress. **b** Response to abiotic stress. **c** Response to biotic stress. **d** Response to salt stress. **e** Response to osmotic stress. **f** Response to oxidative stress. The DEGs of fold change ≥2 were compared and analyzed

As described above and previous studies, treatment with HCN at lower concentrations could enhance plant stress tolerance [12, 25]; thus, the DEGs related to stress response were analysed. As shown in Fig. 9a, a total of 315 DEGs (|log_2_ FC| ≥1) related to stress (GO:0006950) were found in HCN-treated samples, while a total of 208 DEGs (|log_2_ FC| ≥1) were found in ET-treated samples. Further analysis showed that a total of 203 and 92 DEGs in HCN-treated samples were assigned to abiotic stress (GO:0009628) and biotic stress (GO:0009607) (Fig. 9b, c), respectively, indicating that HCN plays a more important role in plant response to abiotic stress than biotic stress. In ET-treated samples, a total of 141 and 60 DEGs were assigned to abiotic stress and biotic stress, respectively (Fig. 9b, c). A total of 117 DEGs related to stress were commonly regulated by HCN and ET. Of these, a total of 80 DEGs related to abiotic stress and 27 DEGs related to biotic stress were regulated by both HCN and ET (Fig. 9b, c). Further analysis revealed that, with HCN treatment, a total of 54 DEGs related to salt stress (GO:0009651), 61 DEGs related to osmotic stress (GO:0006970) and 75 DEGs related to oxidative stress (GO:0006979) were detected (Fig. 9d-f). In comparison, a total of 35, 36 and 42 DEGs related to salt stress, osmotic stress and oxidative stress were detected in ET-treated samples, respectively. There were 20 DEGs (salt stress), 21 DEGs (osmotic stress) and 30 DEGs (oxidative stress) regulated by both HCN and ET.

The top 10 commonly upregulated DEGs related to stress by HCN and ET are shown in Additional file 1: Table S6. Among them, it is interesting to note that the stress-related gene with the highest induced expression level regulating by both HCN and ET was *AT3G09440* (heat shock protein 70–3, HSP70–3), which belongs to HSP70 family proteins and is essential for plant development and plant resistance to environmental stress [26]. In addition to HSP70–3, six more HSP protein transcripts were induced by both HCN and ET (Additional file 1: Table S6). Moreover, the gene expression of UDP-Glycosyltransferase superfamily protein (*UDP72B1*), which is involved in metabolizing xenobiotica (chloroaniline and chlorophenol) and salt stress responses [27, 28], was significantly induced by HCN (fold change = 5.33) and ET (fold change = 2.75) (Additional file 1: Table S6).

As shown in Fig. 9, there were 198 DEGs related to stress that were significantly induced by HCN but not by ET. Of these, the top 10 DEGs included the *AT1G56600* (galactinol synthase 2, GOLS2), *AT3G22840* (Chlorophyll A-B binding family protein, ELIP1), and *AT5G36910* (Thionin 2.2, THI2.2), where the expressions were upregulated by 15-fold, 10-fold and 3.7-fold, respectively, when compared to the CK (Additional file 1: Table S7). Interestingly, there were 91 DEGs related to stress that were significantly induced by ET but not by HCN. Among them, the gene with the highest expression level induced by ET was *AT3G11340* (UDP-Glycosyltransferase superfamily protein, UGT76B1), which showed approximately 13-fold higher than the CK (Additional file 1: Table S7). These data indicated that HCN and ET play different roles in regulating plant responses to environmental stress, apart from the possible synergistic relationship.

## Discussion

Since the confirmation that HCN is formed as a co-product during ET biosynthesis, questions have been raised regarding the physiological significance of this metabolite in plants [9]. It has been proposed that HCN may be a phytotoxic agent at higher concentration and may have a regulatory function at lower concentration [2]. However, the toxic or regulatory function for HCN in plant metabolism remains controversial. In the present study, RNA-seq method was used to reveal the regulatory role of HCN in *Arabidopsis thaliana* and the significant differences in the regulation of gene expression between HCN- and ET-treated seedlings. The results showed that a total of 1305 DEGs and 918 DEGs were significantly (|log_2_ FC| ≥1) regulated by HCN and ET, respectively. A total of 474 genes (|log_2_ FC| ≥1) were commonly regulated by HCN and ET. These findings, in agreement with previous studies, indicate that HCN is possibly an important signalling molecule involved in the control of different metabolic, physiological and developmental processes in plants, rather than a waste co-product of ET biosynthesis [2, 9]. Since a large number of genes were co-regulated by HCN and ET, suggesting that there may be a synergistic relationship in the regulation of cellular processes between them, as previous observations have shown that they elicit some similar physiological responses including alleviation of seed dormancy and enhancement of plant resistance to environmental stress [14, 22].

According to the results of GO and KEGG analysis, we found that the DEGs regulated by HCN and ET were mainly enriched in plants response to stimuli and hormones (Figs. 6 and 7). Interestingly, HCN and ET co-regulated DEGs were mainly associated with auxin signalling transduction, including the family genes from *SAURs*, *AUX/IAA* and *GH3s* (Additional file 1: Table S3). Auxin is identified as a plant growth hormone that plays a critical role in leaf growth and development and is also implicated in plant defence signalling pathways [29, 30]. SAUR genes are rapidly upregulated in response to auxin; however, their functions remain elusive [29]. In comparison, more functions have been uncovered about the *Aux/IAA* family genes. It has been demonstrated that *Aux/IAA* genes are required for stress tolerance. The Aux/IAA proteins are auxin-sensitive repressors that mediate diverse physiological and developmental processes in plants [31, 32]. Shani et al. (2017) reported that promoting the transcription of *IAA5*, *IAA6* and *IAA19* resulted in enhanced tolerance to abiotic stress, whereas recessive mutations in these *IAA* genes resulted in decreased tolerance to stress conditions [33]. Interestingly, our RNA-seq data showed that, in addition to *IAA19* gene (fold change = 1.8), *IAA6* and *IAA17* were upregulated 3.2-fold and 2.1-fold by HCN, respectively (Additional file 1: Table S4). Thus, these findings encourage us to determine whether these genes contribute to HCN-mediated plant stress tolerance in the future.

In addition to auxin signaling pathway, our data showed that the genes regulated by HCN were also involved in ABA, GA, and BR signalling pathway (Additional file 1: Table S3). It has been demonstrated that the phytohormone ABA serves as an endogenous messenger in the biotic and abiotic stress responses of plants [34]. Under non-stress conditions, ABA is also required to fine-tune growth and development. It is evident that ET can cross-talk with ABA pathways either antagonistically or synergistically to regulate plant development and stress adaptation. Additionally, HCN may engage in cross-talk with ABA pathways antagonistically because the *HAB1* (fold change = 2.04), *PYL1* (PYR/PYL/RCAR family proteins; fold change = 1.67) and *ABI2* (PP2C family protein; fold change = 1.57) were upregulated by HCN (Additional file 1: Table S3). However, the relationships between HCN, ET, and ABA should be further investigated to confirm their functions in plant growth and development under certain conditions.

It is noteworthy that the gene expression of *RGL2*, which encodes a DELLA protein, was significantly upregulated by HCN and ET 3.8-fold and 2.5-fold higher than the CK, respectively. DELLA proteins act as repressors of GA-dependent processes to retard plant growth [35]. However, under stress conditions, deceleration of the growth is regarded as one of the strategies that help to improve the survival of plants [36, 37]. In addition, it is likely that the ability to reduce cell growth under unfavourable conditions may not only allow conservation of energy for defence purposes but also limit the risk of heritable damage [2]. ET signalling was found to provide salt stress and cold stress tolerance by enhancing the function of DELLAs [38, 39]. Therefore, it seems that at least part of the growth regulatory action and stress acclimation of HCN is through cross-talk with DELLA proteins, such as RGL2. DELLA proteins have been demonstrated to interact with multiple hormone pathways and they have been regarded as key components of the plant growth regulation and stress response network [40, 41]. Thus, more research is necessary to uncover whether DELLAs are involved in the interactions of HCN with other hormones in the future.

In this study, parts of genes related to the BR signalling pathway were regulated by both HCN and ET (Additional file 1: Table S3), thus it is worth focusing on the cross-talk among them. BRs are an important group of plant steroid hormones involved in numerous aspects of plant life, including growth, development and response to various stresses [42]. It was shown that BR binds to the extracellular domain of the cell-surface receptor kinase BRASSINOSTEROID INSENSITIVE1 (BRI1) to activate BRI1 kinase activity [43]. BRI1 activation involves the recruitment of the co-receptor kinase BRI1-ASSOCIATED RECEPTOR KINASE1 (BAK1) and disassociation of the inhibitory protein BRI1 KINASE INHIBITOR1 (BKI1). In addition, it was suggested that BSKs are the substrates of BRI1 kinase that activate downstream BR signal transduction such as BRI1-SUPPRESSOR1 (BSU1). BSU1 can activate BRASSINAZOLE RESISTANT1 (BZR1) and BRI1-EMS-SUPPRESSOR1 (BES1) indirectly by inactivating the kinase BRASSINOSTEROID INSENSITIVE2 (BIN2), which is a negative regulator in the BR signalling pathway [44]. However, the interactions between BRs and ET are still largely unknown. Some studies suggest that BRs positively influence ET biosynthesis through the regulation of ACS and ACC oxidase activities [45], whereas how ET affects the BRs synthesis and signalling transduction remain unclear. In the present study, the RNA-seq data showed that gene expression of the *BSK2* was upregulated while the *BKI1*, *BIN2* and *BZR1* were downregulated by HCN and ET when compared to the CK (Additional file 1: Table S3). Consequently, it is likely that both HCN and ET positively affect BR signal transduction to some extent.

Strikingly, the genes associated with ET biosynthetic pathway were downregulated by HCN based on the RNA-seq data in this study (Fig. 8), although several previous studies have demonstrated that seed dormancy removal by 1 mM HCN involves modifications in the ET biosynthetic pathway [13, 15]. However, our findings are consistent with the report of Garcia et al. (2010) that HCN accumulation in *cys-c1* mutant plants showed reduced ET production and repressed expression of several genes related to ET signalling and metabolism, such as *ACS6*, *ERF6*, and *ERF105*, when compared to wild-type plants [21]. Consequently, it seems that the role of HCN in plants does not rely on the ET feedback effect. In fact, our hypothesis is also in agreement with the results of Seo et al. (2011), showing that exogenous cyanide (KCN) but not ET complements blast fungus resistance in *ACS/ACO* knockdown rice plants [11]. In addition, it should be noted that Oracz et al. (2008) stated that the expression of the transcription factor *ERF1* was markedly stimulated by 1 mM HCN during the release of sunflower seed dormancy but, it did not significantly affect ET production or the expression of genes involved in ET biosynthesis or the first steps of the ET signalling pathway [15]. Furthermore, there is no direct evidence that *ERF1* is regulated by HCN or other molecular signals such as ROS during seed germination because a higher concentration of HCN (1 mM) has been shown to promote ROS accumulation apparently [10, 14]. Importantly, our data showed that the transcription of NADPH oxidase (*NADPHox*) genes were not significantly affected by 20 μM HCN treatment. As shown in Additional file 1: Table S5, the fold changes of NADPH/respiratory burst oxidase protein D (*RbohD*) and *RbohF* in CK vs HCN were 0.77 and 1.38, respectively. Consistent with our findings, Arenas-Alfonseca et al. (2018) reported that the effect of HCN on root hair elongation is independent of H_2_O_2_ production and direct NADPH oxidase inhibition [46]. Taken together, we speculate that HCN itself, especially at lower concentrations, should serve as a signalling molecule in plants but more studies are needed to reveal its target proteins (receptors) and its cross-talk with ET under certain conditions.

Our data suggest that a lower concentration of HCN (20 μM) treatment may not significantly affect mitochondrial respiration because there were no significant inhibition of gene expression (|log_2_ FC| ≥1) associated with the mitochondrial respiration chain (Table 4). This result is in agreement with the previous study showed that transient accumulation of HCN in the *cys-c1* mutant did not alter mitochondrial respiration rates in Arabidopsis seedlings [21]. Notably, it has been proposed that HCN is involved in the induction of the AOX gene [2, 16, 47], which mediates cyanide-resistant respiration and whose expression may play positive roles in plant resistance to biotic and abiotic stress [48–50]. However, it appears that the induction of AOX expression by HCN is an indirect effect rather than a direct effect, according to our current data from RNA-seq. Since a variety of studies have demonstrated that the main functions of AOX are to maintain the mitochondrial redox state and decrease the production of ROS [50–52], it is possible that the observed induction in the expression of AOX by HCN under stress conditions might be associated with some other signal molecules, such as ROS, because the transient accumulation of HCN, during an extreme ET burst in stressed tissue, can significantly block cellular respiration and induce large amounts of ROS production [2, 12]. In other words, the regulatory actions of HCN on the plant mitochondrial respiration pathway, depending on its concentration, should be further confirmed by molecular and genetics methods.

In contrast to HCN treatment, ET treatment significantly induced several genes related to the mitochondrial respiratory chain, including cytochrome *c* oxidase and NADH dehydrogenase, indicating that ET has a positive role in the regulation of mitochondrial respiration. Additionally, it should be noted that ET treatment upregulated the gene expression of AOX (HCN resistance gene) rather than CAS (HCN detoxification gene), suggesting that AOX gene is probably regulated by ET but CAS gene is generally regulated by HCN (especially at a higher concentration). Considering that a large amount of HCN is generated along with ET biosynthesis in vivo but this is not the case when ET is applied externally, which may explain why the HCN detoxification genes (CAS family) were not significantly induced by ET treatment. Likewise, the induction of *AOX* by ET probably contributes to the alleviation of respiration inhibition by HCN burst during ET biosynthesis in plants.

In plants, ET has been regarded as a stress-hormone besides its roles in regulation of plant growth and development [53]. Notably, it has been speculated that sub lethal levels of HCN may trigger many events which lead to the acclimation of plants growing under adverse conditions [2], although the mechanism is still unclear. In this study, we found that a total of 117 DEGs related to stress were co-regulated by HCN and ET (Fig. 9), demonstrating that HCN and ET may synergistically regulate plant stress response. Moreover, the data showed that there were 198 stress-related DEGs were significantly induced by HCN but not by ET, thus it is necessary to further investigate who is the key component of HCN-induced plant stress resistance in the future. It is noteworthy to mention that *GolS2*, whose over-expression has been shown to increase plant tolerance to salt, chilling, and high-light stress [54–56], was upregulated by 15-fold by HCN when compared to the CK. Taji et al. (2002) stated that overexpression of *GolS2* in transgenic Arabidopsis caused an increase in endogenous galactinol and raffinose, and showed reduced transpiration from leaves to improve drought tolerance [55]. The results from Sengupta et al. (2015) proposed that the stress-inducible GolS2 plays a key role in the accumulation of galactinol and raffinose under abiotic stress conditions, which may function as osmoprotectants in drought-stress tolerance of plants [57]. Similarly, Selvaraj et al. (2017) reported that over-expression of *AtGolS2* was able to confer drought tolerance and increase grain yield in two different rice (*Oryza sativa*) genotypes under dry field conditions [58]. Given that GolS2 is involved in stress acclimation and markedly responds to HCN treatment, it appears that *GolS2* is probably one of the key candidate genes contributing to HCN-mediated plant stress adaptation. Similarly, it is noteworthy that the gene of *THI2.2*, whose expression was markedly induced by HCN (3.7-fold) but not by ET, might be another key gene that is associated with HCN-induced plant pathogen resistance. It has been demonstrated that THI2.2 is expressed at a low basal level in seedlings and rosette leaves, encodes a PR (pathogenesis-related) protein and belongs to the PR-13 family [59, 60]. However, the function of THI2.2 remains unclear although it was predicted to be involved in the defence response. Interestingly, it was shown that salicylate, ethephon, methyl jasmonate, and silver nitrate did not affect the transcript level of the *THI2.2* gene [60], which is consistent with our findings that ET treatment did not induce its transcript at all. In this case, it is necessary to study whether THI2.2 is a key member of HCN-induced plant disease resistance in the future.

Recently, Garcia et al. (2019) reported that the altered immune response observed in the HCN accumulated Arabidopsis mutant (*cas-c1*) through posttranslational modification of proteins by S-cyanylation, which is involved in the regulation of primary metabolic pathways, such as glycolysis, and the Calvin and S-adenosylmethionine cycles [61]. Of these, a set of 163 proteins susceptible to S-cyanylation included the PEPTIDYL-PROLYL CIS-TRANS ISOMERASE 20–3 (CYP20–3) and ENOLASE 2 (ENO2). Here, we found that *CYP20–3* and *ENO2* were induced but not significantly by HCN, where the expression levels were approximately 1.47-fold and 1.43-fold higher than those in CK (Additional file 1: Table S8). In addition, HCN treatment regulated a large number of DEGs enriched in the cysteine and methionine metabolism pathways (Fig. 7c; Additional file 1: Table S9). However, more experiments are required to uncover how HCN participates in the regulation of these primary carbon metabolisms, and the identification of S-cyanylated proteins should be further considered as it is beneficial for elucidating the HCN signalling mechanism [61].

In addition to the above-mentioned genes, another striking gene regulated by HCN that should be mentioned is *AT2G15020*, whose gene expression was upregulated by 23-fold. However, the function of the AT2G15020 is unknown currently. Therefore, further research is needed in the future to decipher the role of AT2G15020 in plants and its possible mechanism of responding to HCN.

## Conclusion

In this study, we focused on the regulation of gene expression in Arabidopsis by HCN and ET treatment. The transcriptome sequencing data indicated that HCN should be recognized as an important signal molecule, rather than being simply considered a toxic by-product of ET biosynthesis. Here, we found that a large number of genes were regulated by HCN, and some of these genes were co-regulated with ET. The DEGs of CK vs HCN were associated with plant growth and development and plant response to stress. In addition, HCN-induced gene expression might be, at least partly, shared with other plant hormone signal transduction pathways. However, the cross-talk between HCN, ET and other hormones is required to perform further validation experiments with some key genes discovered in this study.

In addition, HCN and ET are small gaseous molecules with similar chemical properties and simple structures that are generated at the same time. However, there was no experimental data, including the data from the RNA-seq determination in this study, indicating that HCN shares the same receptor(s) as ET. Since HCN is a simple, small and diffusible molecule, it is highly improbable that its transduction involves specific receptor(s) [2]. Thus, the question arises as to what might be the receptor(s) of HCN and how HCN affects gene expression in plants. Genetic analysis and further physiological studies will improve our understanding of how HCN is perceived and transduced into specific downstream responses.

## Methods

### Plant materials and treatments

The Arabidopsis Columbia ecotype (Col-0) seeds used in this study were obtained from Arabidopsis Biological Resource Center. For growth under sterile conditions, seeds were surface sterilized with 25% (*v*/v) commercial bleach, followed by six washes with sterile distilled water. The seeds were sown onto half-strength Murashige and Skoog (MS)-containing 0.8% agar plates with 10 g/L sucrose. Seedlings were transferred to soil and grown in growth chambers with 16 h of light (approximately 120 μmol m^− 2^ s^− 1^) at 22 °C, 8 h of dark at 18 °C, and 70% relative humidity. For transcriptome analysis, approximately 4-weeks old seedlings were used for the following treatments.

HCN treatment was carried out according to the method of Bogatek and Lewak (1988) with some modification [62]. Arabidopsis seedlings were placed in a glassy closed container (20 L) and the HCN inside was released from another closed round-bottom flask where the HCN was produced by acidifying the 10 mL 1 mM KCN solution with 10 mL of lactic acid (10%, v/v). The HCN contents were detected with a cyanide gas detector (GT-901), which has been calibrated before use. The reaction was terminated while the final concentration of cyanide in glassy container reached 20 μM HCN, which is a relatively lower concentration and we found this concentration helps to improve the plants tolerance to environmental stress [12].

ET treatment was carried out according to the method of Khan et al. (2008) with some modification [63]. Arabidopsis seedlings were placed in a glassy closed container (20 L) and then the ET donor: 500 ppm ethephon (2-chloro-ethylphosphonic acid) was applied to generate ET gas. The control seedlings were placed in a similar size of glassy closed container (20 L). After all materials exposure to light (120 μmol m^− 2^ s^− 1^), 22 °C for 2 h, the containers were opened and gaseous HCN and ET released. Then the HCN-treated, ET-treated and the control seedlings were collected for the total RNA extraction.

### RNA extraction and Illumina sequencing

Total RNA was extracted using an EasyPure Plant RNA Kit (Transgene Co., China) and at least 5 individual seedlings of each treatment were homogenized with liquid nitrogen, and three biological replicates for each treatment were carried out in this study. The RNA quantities were analyzed by an Agilent 2100 Bioanalyzer (Agilent Technologies, Santa Clara, CA, USA). Equal quantities of total RNA from the three biological replicates were pooled before used for cDNA library construction and sequencing. cDNA libraries were constructed using the Illumina RNA-seq kit (Illumina, USA). Solexa adapters were then ligated to the ends of the cDNA fragments for Solexa sequencing. High-throughput sequencing was performed using Illumina Hiseq 2500 (Illumina, USA). Reads were 125 bases in length and generated from each end of the DNA fragments in paired-end sequencing.

### Gene annotation and differential expression analysis

The adaptor sequences and low-quality sequence reads were removed from the data sets. Raw sequences were transformed into clean tags after data processing. Gene Ontology (GO; http://www.geneontology.org/) and Kyoto Encyclopedia of Genes and Genomes (KEGG; www.kegg.jp) analyses were subsequently performed. GO is an internationally standardized gene function classification system for comprehensively describing the properties of genes and their products in any organism. The basic unit of GO is the GO term and each GO term belongs to a type of ontology. In gene expression profiling analysis, GO enrichment analyses of functional significance was performed using hypergeometric testing to map all differentially expressed genes to terms in the GO database by looking for GO terms that were significantly enriched in a given differentially expressed genes (DEGs) relative to the genome background. According to GO analysis, all unigenes were divided into three groups: molecular function (MF), cellular component (CC) and biological process (BP).

KEGG is the major public pathway-related database. Different genes usually cooperate with each other to exercise their biological functions. Pathway-based analysis helps the user to further understand the biological functions of specific genes. Pathway enrichment analysis identifies significantly enriched metabolic pathways and signal transduction pathways in DEGs by comparing them to the whole-genome background [64].

The expression level of each gene was measured as the normalized number of matched clean tags. The normalization method of reads per kilobase per million mapped (RPKM) was used in this study. The false discovery rate (FDR) method was used to tune the threshold *P* value.

### Real-time quantitative PCR analysis

In order to validate the results from transcriptome sequencing analysis, part of genes were confirmed by quantitative real-time PCR (qRT-PCR). All the Primers are listed in Additional file 1: Table S1. qRT-PCR reactions were prepared with the SYBR Green Master Mix Reagent (Applied Biosystems), following the manufacturer’s instruction. Reactions were carried out in Applied Real-Time System (ABI7500). The thermal cycling profile consisted of an initial denaturation at 95 °C for 30 s, 40 cycles at 95 °C for 5 s, and 60 °C for 30 s. All samples were performed in triplicate and relative expression levels were calculated using the delta-delta Ct method of the system. In this study, *ACTIN2* (AT3G18780) gene was used as internal control.

## Additional file

Additional file 1: **Table S1.** All primers for qRT-PCR. **Figure S1.** qRT-PCR analysis of DEGs from CK vs HCN and CK vs ET. (**a**) The top 5 genes co-regulated by HCN and ET were determined by qRT-PCR. The top 5 genes exclusively regulated by HCN (**b)** or ET (**c)** were determined by qRT-PCR. Expression ratios (FPKM fold change) obtained from transcriptome data (green) and qRT-PCR (red). (**d**) Lineage analysis between the transcriptome and qRT-PCR. **Figure S2.** Heatmap of co-regulated genes by HCN and ET. (a) Heatmap of all common DEGs between CK vs HCN and CK vs HCN. (b) Number of DEGs co-regulated by HCN and ET. (c) Heatmap of common DEGs (fold change ≥2) between CK vs HCN and CK vs ET. (d) Number of DEGs (fold change ≥2) co-regulated by HCN and ET. **Table S2.** Common GO terms enriched in CK vs HCN and CK vs ET. **Figure S3.** Comparison of the DEGs related to plant hormone signal transduction between CK vs HCN and CK vs ET. (a) Number of DEGs including up-regulated and down-regulated by HCN and ET. (b) Venn diagram for the number of DEGs regulated by HCN and ET. **Table S3.** The co-regulated DEGs related to plant hormone signaling transduction pathway by HCN and ET. **Table S4.** DEGs related to Auxin/IAAs that were regulated by HCN and ET. **Table S5.** DEGs related to ROS production in CK vs HCN. **Table S6.** Top 10 co-upregulated DEGs related to stress by HCN and ET. **Table S7.** Top 10 DEGs related to stress that were exclusively induced by HCN or ET. **Table S8.** Parts of DEGs related to post-translation regulation by S-cyanylation in CK vs HCN. **Table S9.** DEGs enriched in cysteine and methionine metabolism that were regulated by HCN. (DOCX 699 kb)

## Abbreviations

ABA: Abscisic acid
ACC: 1-amino-cyclopropane-1-carboxylate synthase
ACO: 1-amino-cyclopropane-1-carboxylate oxidase
AOX: Alternative oxidase
AUX/IAA: AUXIN/INDOLE ACETIC ACID
BP: Biological process
BR: Brassinosteroid
CC: Cellular component
COX: Cytochrome c oxidase
CTK: Cytokinin
CYP20–3: PEPTIDYL-PROLYL CIS-TRANS ISOMERASE 20–3
DEGs: Differentially expressed genes
ENO2: ENOLASE 2
ERFs: Ethylene response factors
ET: Ethylene
FC: Fold change
FDR: False discovery rate
GA: Gibberellin
GH3s: GRETCHEN HAGEN3s
GO: Gene ontology
HCN: Hydrogen cyanide
HSP 70: Heat shock protein 70
KEGG: Kyoto Encyclopedia of Genes and Genomes
MF: Molecular function
MS: Murashige and Skoog
NADPHox: NADPH oxidase
NO: Nitric oxidase
PP2C: Protein phosphatase 2C
qRT-PCR: Quantitative real-time PCR
RNA-seq: RNA sequencing
ROS: Reactive oxygen species
RPKM: Reads per kilobase per million mapped
SA: Salicylic acid
SAURs: SMALL AUXIN UP RNAs
THI2.2: Thionin 2.2
UDP72B1: UDP-Glycosyltransferase superfamily protein
*β*-CAS: *β*-cyanoalanine synthase
