# Supplementary material for: Comparative transcriptome analysis reveals significant differences in the regulation of gene expression between hydrogen cyanide- and ethylene-treated Arabidopsis thaliana
Source: BMC Plant Biol. 2019 Mar 4;19:92. doi: 10.1186/s12870-019-1690-5 (PMC6399987; doi:10.1186/s12870-019-1690-5)
Supplement: Supplementary file 1 — Table S1. All primers for qRT-PCR. Figure S1. qRT-PCR analysis of DEGs from CK vs HCN and CK vs ET. (a) The top 5 genes co-regulated by HCN and ET were determined by qRT-PCR. The top 5 genes exclusively regulated by HCN (b) or ET (c) were determined by qRT-PCR. Expression ratios (FPKM fold change) obtained from transcriptome data (green) and qRT-PCR (red). (d) Lineage analysis between the transcriptome and qRT-PCR. Figure S2. Heatmap of co-regulated genes by HCN and ET. (a) Heatmap of all common DEGs between CK vs HCN and CK vs HCN. (b) Number of DEGs co-regulated by HCN and ET. (c) Heatmap of common DEGs (fold change ≥2) between CK vs HCN and CK vs ET. (d) Number of DEGs (fold change ≥2) co-regulated by HCN and ET. Table S2. Common GO terms enriched in CK vs HCN and CK vs ET. Figure S3. Comparison of the DEGs related to plant hormone signal transduction between CK vs HCN and CK vs ET. (a) Number of DEGs including up-regulated and down-regulated by HCN and ET. (b) Venn diagram for the number of DEGs regulated by HCN and ET. Table S3. The co-regulated DEGs related to plant hormone signaling transduction pathway by HCN and ET. Table S4. DEGs related to Auxin/IAAs that were regulated by HCN and ET. Table S5. DEGs related to ROS production in CK vs HCN. Table S6. Top 10 co-upregulated DEGs related to stress by HCN and ET. Table S7. Top 10 DEGs related to stress that were exclusively induced by HCN or ET. Table S8. Parts of DEGs related to post-translation regulation by S-cyanylation in CK vs HCN. Table S9. DEGs enriched in cysteine and methionine metabolism that were regulated by HCN. (DOCX 699 kb) [file 12870_2019_1690_MOESM1_ESM.docx]

**Additional file 1**

**Table S1** All primers for qRT-PCR

| **Gene id** | **Primer (F)** | **Primer (R)** |
| --- | --- | --- |
| AT2G15020 | GTCATTCGTTGGGGTTTTCG | CGTGGATGCGTATGTTGTCTA |
| AT3G09440 | GCTGCTATTGCTTACGGTCT | TCGGGTTTCCACTAATGTCC |
| AT4G03060 | GGAGGTAAAGACCAAAGATGAG | CGACCAGCTTCTGAGTGATAG |
| AT1G74310 | GACGCTGAAGGCAAAGTGAT | CTTGGCTCCGCAACATAGAC |
| AT2G46790 | GGAAGTGGTGCTCAGGCTAT | ATGTGATTCGGTTGGTGTCT |
| AT1G56600 | GGGTCTTGCTAAGGGGCTAA | CCACGAACTCCCAGATACGA |
| AT5G37990 | TGGAAGAGCACTTTGGGAGT | TGACGCTTGAGGAGGATGAA |
| AT1G70260 | CCCCTATTCCGTCTTGTATC | AAACGACTATCGGTGAACTGT |
| AT3G22840 | GTCCAAGTCCAAAGGTATCA | CCCGAAGTTTCAAAATGTCA |
| AT3G25190 | TCAGAAACAAACAGCCCAAGG | TACAAGCACCCGCAACAAGG |
| AT3G11340 | GTGATTGTTGACGCTCTTTGG | TACGGAATTTGTTGCTTGCT |
| AT4G25490 | ACTTCGCTGACTCGGCTTGG | TAAAACGCACCTTCGCTCTG |
| AT1G61255 | ATGGCTACAAAACTCAATCTCC | ACATCACCGTTAGTCTCACCC |
| AT4G22470 | ATAACCATTCCGTTTACTGC | GAACATATTCCAACCAGAGG |
| AT5G05340 | TCAAGCGGCAGCGAATAG | CGGACGATGGAGTCAGTAGAG |


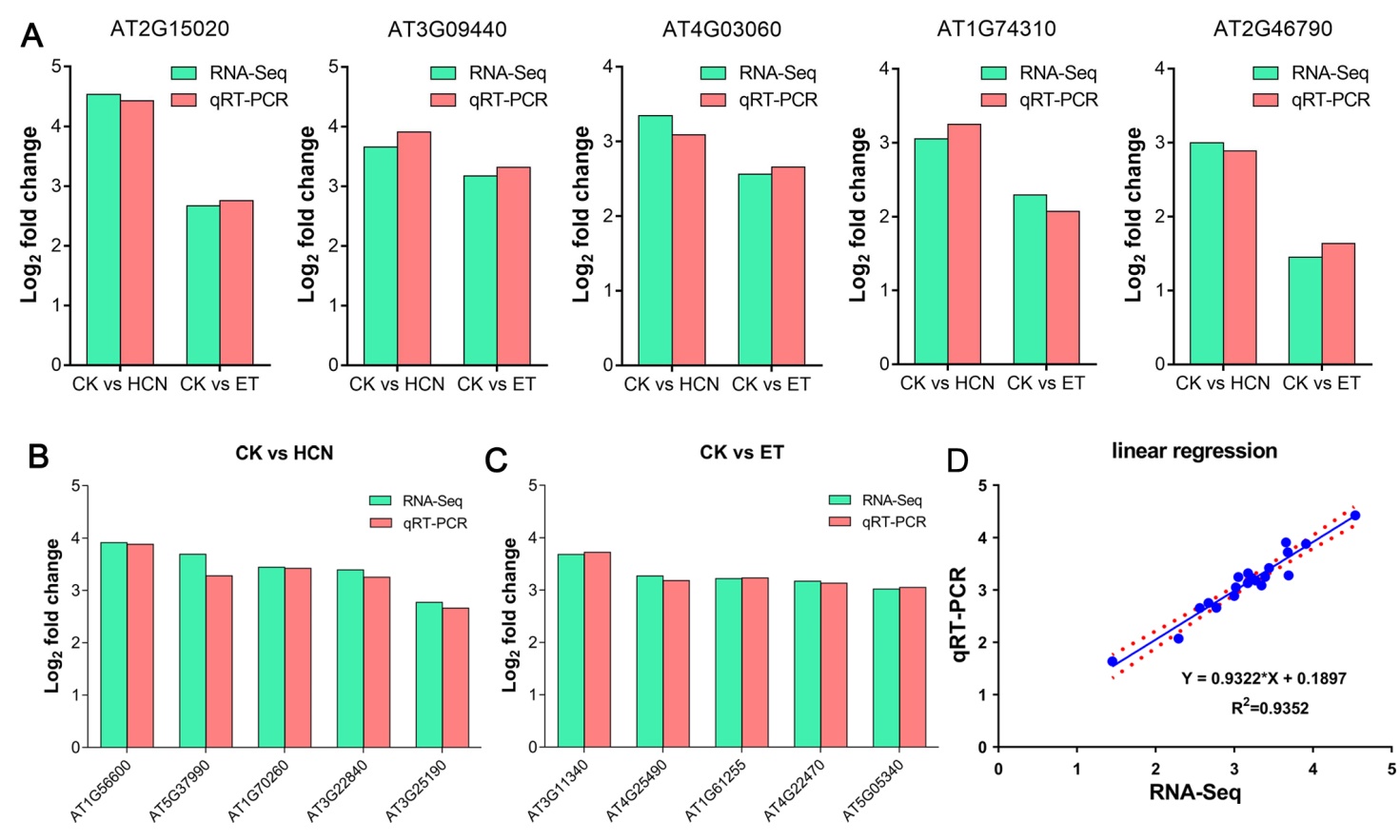


**Figure S1.** qRT-PCR analysis of DEGs from CK vs HCN and CK vs ET. (**a)** The top 5 genes co-regulated by HCN and ET were determined by qRT-PCR. The top 5 genes exclusively regulated by HCN (**b)** or ET (**c)** were determined by qRT-PCR. Expression ratios (FPKM fold change) obtained from transcriptome data (green) and qRT-PCR (red). (**d**) Lineage analysis between the transcriptome and qRT-PCR.


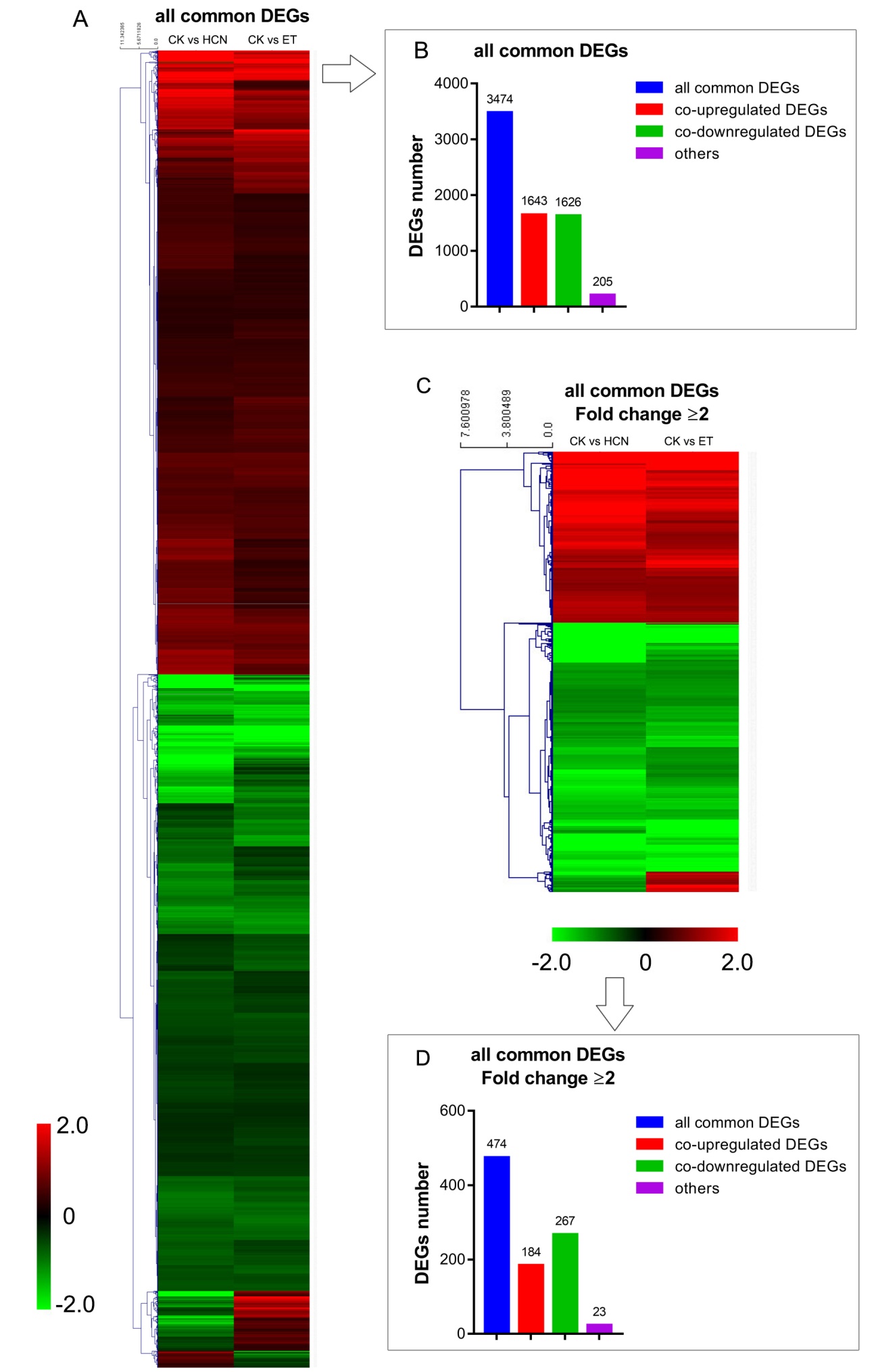


**Figure S2** Heatmap of co-regulated genes by HCN and ET. **(a)** Heatmap of all common DEGs between CK vs HCN and CK vs HCN. (**b**) Number of DEGs co-regulated by HCN and ET. (**c)** Heatmap of common DEGs (fold change ≥2) between CK vs HCN and CK vs ET. (**d**) Number of DEGs (fold change ≥2) co-regulated by HCN and ET.

**Table S2** Common GO terms enriched in CK vs HCN and CK vs ET

| **GO id** | **GO Term** | **CK vs HCN** | |  | **CK vs ET** | |
| --- | --- | --- | --- | --- | --- | --- |
|  |  | **DEGs** | **FDR** |  | **DEGs** | **FDR** |
| GO:0050896 | response to stimulus | 1864 | 2.24E-16 |  | 1477 | 2.04E-15 |
| GO:0006950 | response to stress | 1118 | 6.43E-09 |  | 886 | 1.62E-08 |
| GO:0042221 | response to chemical | 930 | 7.10E-20 |  | 735 | 9.12E-16 |
| GO:0009628 | response to abiotic stimulus | 742 | 1.45E-27 |  | 570 | 3.65E-20 |
| GO:0010033 | response to organic substance | 639 | 8.48E-17 |  | 498 | 3.93E-12 |
| GO:0044281 | small molecule metabolic process | 601 | 0.003309 |  | 468 | 0.018717 |
| GO:1901700 | response to oxygen-containing compound | 565 | 7.79E-16 |  | 441 | 1.44E-11 |
| GO:0009719 | response to endogenous stimulus | 553 | 3.20E-15 |  | 436 | 5.81E-12 |
| GO:0009725 | response to hormone | 501 | 6.81E-13 |  | 389 | 3.52E-09 |
| GO:0001101 | response to acid | 410 | 1.12E-09 |  | 338 | 1.34E-10 |
| GO:0070887 | cellular response to chemical stimulus | 399 | 7.27E-06 |  | 312 | 0.000169 |
| GO:0006082 | organic acid metabolic process | 359 | 0.000841 |  | 288 | 0.000872 |
| GO:0010035 | response to inorganic substance | 357 | 3.72E-06 |  | 287 | 6.45E-06 |
| GO:0043436 | oxoacid metabolic process | 357 | 0.000974 |  | 288 | 0.00062 |
| GO:0071310 | cellular response to organic substance | 341 | 1.08E-05 |  | 270 | 0.000083 |
| GO:0019752 | carboxylic acid metabolic process | 333 | 0.006196 |  | 265 | 0.009532 |
| GO:0071495 | cellular response to endogenous stimulus | 288 | 4.27E-05 |  | 241 | 1.75E-06 |
| GO:0032870 | cellular response to hormone stimulus | 278 | 5.90E-05 |  | 235 | 9.81E-07 |
| GO:0009314 | response to radiation | 276 | 4.97E-11 |  | 210 | 7.67E-07 |
| GO:0009416 | response to light stimulus | 269 | 7.27E-13 |  | 206 | 2.13E-08 |
| GO:0009755 | hormone-mediated signaling pathway | 260 | 0.000143 |  | 225 | 2.84E-07 |
| GO:0033993 | response to lipid | 247 | 0.000107 |  | 193 | 0.001513 |
| GO:0009266 | response to temperature stimulus | 245 | 3.24E-17 |  | 182 | 1.23E-09 |
| GO:0006970 | response to osmotic stress | 216 | 1.13E-06 |  | 153 | 0.018924 |
| GO:0097305 | response to alcohol | 209 | 0.000571 |  | 167 | 0.001452 |
| GO:0009651 | response to salt stress | 191 | 2.76E-05 |  | 141 | 0.013154 |
| GO:0009737 | response to abscisic acid | 182 | 0.005122 |  | 150 | 0.001895 |
| GO:0010038 | response to metal ion | 177 | 0.008807 |  | 142 | 0.012815 |
| GO:0006979 | response to oxidative stress | 176 | 0.000327 |  | 148 | 4.33E-05 |
| GO:0042592 | homeostatic process | 170 | 3.08E-05 |  | 128 | 0.005129 |
| GO:0009617 | response to bacterium | 153 | 0.004676 |  | 128 | 0.001007 |
| GO:0009409 | response to cold | 147 | 9.27E-08 |  | 100 | 0.016003 |
| GO:0009733 | response to auxin | 138 | 0.00173 |  | 118 | 0.000154 |
| GO:0046686 | response to cadmium ion | 136 | 0.013079 |  | 116 | 0.001407 |
| GO:0042742 | defense response to bacterium | 128 | 0.011783 |  | 104 | 0.013154 |
| GO:1901698 | response to nitrogen compound | 121 | 7.42E-06 |  | 88 | 0.007284 |
| GO:0009415 | response to water | 108 | 0.023809 |  | 93 | 0.002892 |
| GO:0009414 | response to water deprivation | 107 | 0.012679 |  | 93 | 0.000935 |
| GO:0009408 | response to heat | 106 | 3.49E-09 |  | 91 | 1.2E-09 |
| GO:0019725 | cellular homeostasis | 101 | 0.005122 |  | 79 | 0.028169 |
| GO:0006457 | protein folding | 100 | 0.000581 |  | 88 | 2.34E-05 |
| GO:0009611 | response to wounding | 94 | 0.000125 |  | 85 | 9.81E-07 |
| GO:0010243 | response to organonitrogen compound | 92 | 2.08E-05 |  | 68 | 0.005953 |
| GO:0009753 | response to jasmonic acid | 90 | 2.02E-05 |  | 70 | 0.000757 |
| GO:0009751 | response to salicylic acid | 89 | 0.014436 |  | 72 | 0.022608 |
| GO:0009639 | response to red or far red light | 87 | 0.000404 |  | 65 | 0.021796 |
| GO:0009657 | plastid organization | 84 | 1.47E-06 |  | 58 | 0.012582 |
| GO:0071365 | cellular response to auxin stimulus | 83 | 0.005767 |  | 85 | 4.82E-08 |
| GO:0009734 | auxin-activated signaling pathway | 80 | 0.005767 |  | 85 | 4.11E-09 |
| GO:0080167 | response to karrikin | 75 | 3.11E-08 |  | 58 | 1.38E-05 |


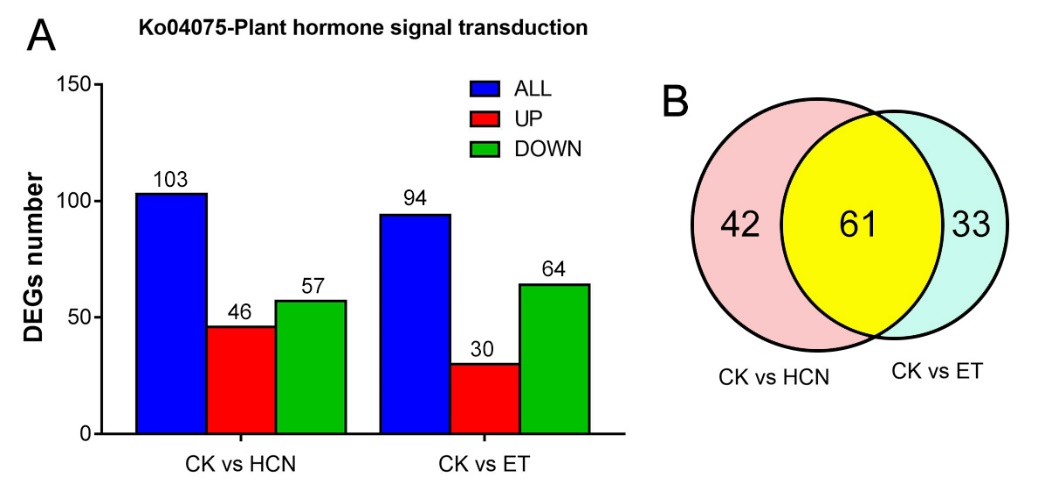


**Figure S3** Comparison of the DEGs related to plant hormone signal transduction between CK vs HCN and CK vs ET. **(a)** Number of DEGs including up-regulated and down-regulated by HCN and ET. (**b)** Venn diagram for the number of DEGs regulated by HCN and ET.

**Table S3** The co-regulated DEGs related to plant hormone signaling transduction pathway by HCN and ET

| **Gene id** | **Gene annotation** | **CK vs HCN** | **CK vs ET** |
| --- | --- | --- | --- |
|  |  | **Fold Change** | **Fold Change** |
| AT4G03400 | DFL2, auxin-responsive GH3 family protein | 2.63  2.36  2.28  2.13  2.04  1.78  1.59  1.55  0.7 | 2.15  1.65  1.58  0.63  1.28  0.74  0.49  0.73  0.61 |
| AT2G21210 | SAUR6, small auxin upregulated RNA 6 |  |  |
| AT4G38860 | SAUR16, small auxin upregulated RNA 16 |  |  |
| AT5G18020 | SAUR20, small auxin upregulated RNA 20 |  |  |
| AT4G38840 | SAUR14, small auxin upregulated RNA 14 |  |  |
| AT3G15540 | IAA19, indole-3-acetic acid inducible 19 |  |  |
| AT5G18030 | SAUR21, small auxin upregulated RNA 21 |  |  |
| AT2G21050 | LAX2, like AUXIN RESISTANT 2 |  |  |
| AT2G46690 | SAUR32, small auxin upregulated RNA 32 |  |  |
| AT3G23030 | IAA2, indole-3-acetic acid inducible 2 | 0.66 | 0.58 |
| AT2G38120 | AUX1, AUXIN RESISTANT 1 | 0.65 | 0.4 |
| AT4G14560 | IAA1, indole-3-acetic acid inducible 1 | 0.6 | 0.65 |
| AT4G34790 | SAUR3, small auxin upregulated RNA 3 | 0.54 | 0.56 |
| AT5G43700 | IAA4, indole-3-acetic acid inducible 4 | 0.52 | 0.43 |
| AT4G32280 | IAA29, indole-3-acetic acid inducible 29 | 0.43 | 0.21 |
| AT3G62100 | IAA30, indole-3-acetic acid inducible 30 | 0.16 | 0.22 |
| AT2G23170 | GH3.3, Auxin-responsive GH3 family protein | 1.5 | 2.09 |
| AT3G53250 | SAUR57, small auxin upregulated RNA 57 | 0.56 | 0.67 |
| AT5G18080 | SAUR24, small auxin upregulated RNA 24 | 1.77 | 0.54 |
| AT4G34760 | SAUR50, small auxin upregulated RNA 50 | 1.33 | 0.73 |
| AT3G03820 | SAUR29, small auxin upregulated RNA 29 | 1.65 | 0.48 |
| AT5G18010 | SAUR19, small auxin upregulated RNA 19 | 1.5 | 0.3 |
| AT3G12830 | SAUR72, small auxin upregulated RNA 72 | 1.39 | 1.95 |
| AT4G27260 | GH3.5, Auxin-responsive GH3 family protein | 0.79 | 0.49 |
| AT1G72770 | HAB1, hypersensitive to ABA1 | 2.04 | 1.41 |
| AT5G46790 | PYL1, pyrabactin resistance like protein; ABA sensor | 1.67 | 1.43 |
| AT5G57050 | ABI2, protein phosphatase 2C family protein | 1.57 | 1.44 |
| AT1G45249 | ABF2, abscisic acid responsive elements-binding factor 2 | 1.41 | 1.37 |
| AT2G38310 | PYL4, pyrabactin resistance like protein; ABA sensor | 1.39 | 1.35 |
| AT5G53160 | PYL8, a regulatory component of ABA receptor | 0.71 | 0.65 |
| AT3G19290 | ABF4, abscisic acid-responsive elements binding factor 4 | 0.69 | 0.63 |
| AT5G63650 | SRK2H, SNF1-related protein kinase 2.5; SNRK2.5 | 0.64 | 0.72 |
| AT5G05440 | PYL5, function as abscisic acid sensor | 0.54 | 0.7 |
| AT5G66880 | SRK2I, sucrose nonfermenting 1(SNF1)-related protein kinase 2.3 | 0.74 | 0.75 |
| AT2G23030 | SRK2J, SNF1-related protein kinase 2.9 (SNRK2.9) | 0.34 | 0.45 |
| AT5G62920 | ARR6, cytokinin response regulator 6 | 1.98 | 1.58 |
| AT3G16857 | ARR1, Arabidopsis response regulator 1; response to cytokinin | 0.7 | 0.65 |
| AT3G48100 | ARR5, Arabidopsis response regulator 5; response to cytokinin | 1.37 | 1.41 |
| AT2G01760 | ARR14, Arabidopsis response regulator 14; response to cytokinin | 0.77 | 0.73 |
| AT3G57040 | ARR9, Arabidopsis response regulator 9; response to cytokinin | 0.8 | 0.5 |
| AT5G46570 | BSK2, brassinosteroid signaling kinase 2 | 1.57 | 1.45 |
| AT5G42750 | BKI1, BRI1 kinase inhibitor 1 | 0.61 | 0.6 |
| AT4G18710 | BIN2, brassinosteroid-insensitive 2 | 0.76 | 0.71 |
| AT3G50070 | CYCD3-3, cyclin-dependent protein serine/threonine kinase activity | 1.35 | 1.3 |
| AT1G75080 | BZR1, brassinazole resistant 1 | 0.79 | 0.78 |
| AT3G03450 | RGL2, encodes a DELLA protein | 3.87 | 2.52 |
| AT3G63010 | GID1B, GA INSENSITIVE DWARF1B | 0.49 | 0.6 |
| AT5G17490 | RGL3, RGA-like protein 3; DELLA subfamily member | 0.47 | 0.64 |
| AT1G66350 | RGL1, RGA-like 1; Negative regulator of GA responses | 1.31 | 1.33 |
| AT2G25490 | EBF1, EIN3-binding F box protein 1 | 1.95 | 1.97 |
| AT5G47220 | ERF2, ethylene responsive element binding factor 2 | 0.68 | 1.76 |
| AT3G20770 | EIN3, ethylene insensitive 3 | 0.79 | 0.75 |
| AT5G03280 | EIN2, ethylene insensitive 2 | 0.8 | 0.67 |
| AT5G25350 | EBF2, EIN3-binding F box protein 2 | 1.29 | 1.32 |
| AT1G22070 | TGA3, transcription factor TGA3 | 0.6 | 0.75 |
| AT1G19180 | JAZ1, jasmonate-zim-domain protein 1 | 0.48 | 2.16 |
| AT1G70700 | TIFY7, response to jasmonic acid stimulus | 0.31 | 0.43 |
| AT5G57560 | XTH22, xyloglucan endotransglucosylase/hydrolase | 0.29 | 0.53 |
| AT5G44080 | Basic-leucine zipper (bZIP) transcription factor family protein | 0.7 | 0.66 |
| AT1G17380 | TIFY11A, also known as jasmonate-zim-domain protein 5 | 0.57 | 2.13 |
| AT4G19660 | NPR4, NPR1-like protein 4 | 0.79 | 0.72 |

**Table S4** DEGs related to Auxin/IAAs that were regulated by HCN or ET

| **Samples** | **Gene id** | **Gene annotation** | **Fold Change** | **log_2_ Fold Change** | **pval** | **padj** |
| --- | --- | --- | --- | --- | --- | --- |
| CK vs HCN | AT1G52830 | IAA6, indole-3-acetic acid 6 | 3.22 | 1.69 | 0.00015 | 8.72E-04 |
|  | AT1G04250 | IAA17, indole-3-acetic acid 17 | 2.09 | 1.07 | 5.00E-05 | 3.21E-04 |
|  | AT3G15540 | IAA19, indole-3-acetic acid 19 | 1.78 | 0.83 | 5.00E-05 | 3.21E-04 |
|  | AT2G22670 | IAA8, indole-3-acetic acid 8 | 1.52 | 0.60 | 5.00E-05 | 3.21E-04 |
|  | AT1G04550 | IAA12, indole-3-acetic acid 12 | 1.50 | 0.58 | 5.00E-05 | 3.21E-04 |
|  | AT3G23030 | IAA2, indole-3-acetic acid 2 | 0.66 | -0.59 | 5.00E-05 | 3.21E-04 |
|  | AT4G14560 | IAA1, indole-3-acetic acid 1 | 0.60 | -0.74 | 5.00E-05 | 3.21E-04 |
|  | AT5G25890 | IAA28, indole-3-acetic acid 28 | 0.59 | -0.77 | 5.00E-05 | 3.21E-04 |
|  | AT5G43700 | IAA4, indole-3-acetic acid 4 | 0.52 | -0.95 | 5.00E-05 | 3.21E-04 |
|  | AT4G32280 | IAA29, indole-3-acetic acid 29 | 0.43 | -1.20 | 5.00E-05 | 3.21E-04 |
|  | AT2G01200 | IAA32, indole-3-acetic acid 32 | 0.42 | -1.24 | 0.00695 | 2.39E-02 |
|  | AT3G62100 | IAA30, indole-3-acetic acid 30 | 0.16 | -2.63 | 5.00E-05 | 3.21E-04 |
| CK vs ET | AT3G15540 | IAA19, indole-3-acetic acid 19 | 0.74 | -0.43 | 0.013 | 4.92E-02 |
|  | AT2G33310 | IAA13, indole-3-acetic acid 13 | 0.68 | -0.57 | 5.00E-05 | 4.44E-04 |
|  | AT4G14560 | IAA1, indole-3-acetic acid 1 | 0.65 | -0.61 | 5.00E-05 | 4.44E-04 |
|  | AT3G23030 | IAA2, indole-3-acetic acid 2 | 0.58 | -0.77 | 5.00E-05 | 4.44E-04 |
|  | AT3G04730 | IAA16, indole-3-acetic acid 16 | 0.58 | -0.79 | 5.00E-05 | 4.44E-04 |
|  | AT1G04240 | IAA3, indole-3-acetic acid 3 | 0.57 | -0.81 | 5.00E-05 | 4.44E-04 |
|  | AT5G43700 | IAA4, indole-3-acetic acid 4 | 0.43 | -1.21 | 5.00E-05 | 4.44E-04 |
|  | AT3G62100 | IAA30, indole-3-acetic acid 30 | 0.22 | -2.18 | 5.00E-05 | 4.44E-04 |
|  | AT4G32280 | IAA29, indole-3-acetic acid 29 | 0.21 | -2.26 | 5.00E-05 | 4.44E-04 |

**Table S5** DEGs related to ROS production in CK vs HCN

| **Gene id** | **Gene annotation** | **Fold Change** | **log_2_ Fold Change** | **pval** | **padj** |
| --- | --- | --- | --- | --- | --- |
| AT1G64060 | RBOHF, NADPH/respiratory burst oxidase protein F | 1.38 | 0.46 | 0.0013 | 0.005818 |
| AT5G47910 | RBOHD, NADPH/respiratory burst oxidase protein D | 0.77 | -0.36 | 0.0032 | 0.012439 |

**Table S6** Top 10 co-upregulated DEGs related to stress by HCN and ET

| **Gene id** | **Gene annotation** | **CK vs HCN** | | **CK vs ET** | |
| --- | --- | --- | --- | --- | --- |
|  |  | **Fold Change** | **log_2_ Fold Change** | **Fold Change** | **log_2_ Fold Change** |
| AT3G09440 | HSP70-3, Heat shock protein 70 (Hsp 70) family protein | 16.22 | 4.02 | 9.30 | 3.22 |
| AT1G74310 | HSP101, Heat shock protein 101 | 8.29 | 3.05 | 4.89 | 2.29 |
| AT5G52640 | HSP81-1, Heat shock-like protein | 7.53 | 2.91 | 5.27 | 2.40 |
| AT2G20560 | T13C7.15, DNAJ heat shock family protein | 7.49 | 2.90 | 5.73 | 2.52 |
| AT3G12580 | HSP70, Heat shock protein 70 | 7.02 | 2.81 | 4.27 | 2.09 |
| AT5G37670 | HSP15.7, HSP20-like chaperones superfamily protein | 6.16 | 2.62 | 3.63 | 1.86 |
| AT2G26150 | HSFA2, Heat shock transcription factor A2 | 5.83 | 2.54 | 4.02 | 2.01 |
| AT5G56010 | HSP90-3, Heat shock protein | 5.44 | 2.44 | 4.76 | 2.25 |
| AT4G01070 | UGT72B1, UDP-Glycosyltransferase superfamily protein | 5.33 | 2.41 | 2.75 | 1.46 |
| AT3G03190 | GSTF11, Glutathione S-transferase F11 | 5.27 | 2.40 | 3.43 | 1.78 |

**Table S7** Top 10 DEGs related to stress that were exclusively induced by HCN or ET

|  | **Gene id** | **Gene annotation** | **Fold Change** | **log_2_ Fold Change** |
| --- | --- | --- | --- | --- |
| CK vs HCN | AT1G56600 | GOLS2, Galactinol synthase 2 | 15.01 | 3.91 |
|  | AT3G22840 | ELIP1, Chlorophyll A-B binding family protein | 10.46 | 3.39 |
|  | AT3G25190 | VTL5, Vacuolar iron transporter family protein | 6.84 | 2.77 |
|  | AT2G46830 | CCA1, Circadian clock associated 1 | 5.01 | 2.32 |
|  | AT3G09600 | RVE8, Homeodomain-like superfamily protein | 4.70 | 2.23 |
|  | AT5G04950 | NAS1, Nicotianamine synthase 1 | 4.09 | 2.03 |
|  | AT1G21140 | VTL1, Vacuolar iron transporter (VIT) family protein | 4.08 | 2.03 |
|  | AT1G66100 | F15E12.20, Plant thionin | 3.81 | 1.93 |
|  | AT1G54040 | ESP, Epithiospecifier protein | 3.77 | 1.92 |
|  | AT5G36910 | THI2.2, Thionin 2.2 | 3.68 | 1.88 |
| CK vs ET | AT3G11340 | UGT76B1, UDP-Glycosyltransferase superfamily protein | 12.79 | 3.68 |
|  | AT4G25490 | DREB1B, C-repeat/DRE binding factor 1 | 9.63 | 3.27 |
|  | AT5G05340 | PER52, Peroxidase superfamily protein | 8.10 | 3.02 |
|  | AT3G23250 | MYB15, Myb domain protein 15 | 7.51 | 2.91 |
|  | AT1G05680 | UGT74E2, Uridine diphosphate glycosyltransferase 74E2 | 6.95 | 2.80 |
|  | AT4G24570 | PUMP4, Dicarboxylate carrier 2 | 6.85 | 2.78 |
|  | AT5G45340 | CYP707A3, Cytochrome P450, family 707, subfamily A, polypeptide 3 | 6.33 | 2.66 |
|  | AT5G61890 | ERF114, Integrase-type DNA-binding superfamily protein | 6.08 | 2.60 |
|  | AT1G09950 | RAS1, Response to ABA and salt 1 | 5.03 | 2.33 |
|  | AT1G74010 | F2P9.12, Calcium-dependent phosphotriesterase superfamily protein | 5.00 | 2.32 |

**Table S8** Parts of DEGs related to post-translation regulation by S-cyanylation in CK vs HCN

| **Gene_id** | **Gene_name** | **Fold change** | **log_2_ Fold change** |
| --- | --- | --- | --- |
| AT2G36530 | ENO2, enolase 2 | 1.43 | 0.51 |
| AT3G62030 | CYP20-3, PEPTIDYL-PROLYL CIS-TRANS ISOMERASE 20-3 | 1.47 | 0.55 |

Note: The above results are obtained by comparison with the results of Gracia et al. (2019).

**Table S9** DEGs enriched in cysteine and methionine metabolism that were regulated by HCN

| **Gene_id** | **Gene_name** | **Fold change** | **log_2_ Fold change** |
| --- | --- | --- | --- |
| AT5G15950 | SAMDC2, Adenosylmethionine decarboxylase family protein | 4.32 | 2.11 |
| AT3G02020 | AK3, aspartate kinase 3 | 4.22 | 2.08 |
| AT3G22740 | HMT3, homocysteine S-methyltransferase 3 | 1.99 | 0.99 |
| AT4G31990 | ASP5, aspartate aminotransferase 5 | 1.81 | 0.86 |
| AT1G53240 | mMDH1, Lactate/malate dehydrogenase family protein | 1.70 | 0.76 |
| AT4G19710 | AKHSDH2, aspartate kinase-homoserine dehydrogenase II | 1.69 | 0.76 |
| AT3G47520 | MDH, malate dehydrogenase | 1.64 | 0.71 |
| AT3G15020 | mMDH2, mitochondrial malate dehydrogenase 2 | 1.63 | 0.70 |
| AT3G03780 | MS2, methionine synthase 2 | 1.61 | 0.69 |
| AT5G53120 | SPMS, spermidine synthase 3 | 1.58 | 0.66 |
| AT5G28030 | DES1, L-cysteine desulfhydrase 1 | 1.55 | 0.63 |
| AT3G23810 | SAHH2, S-adenosyl-l-homocysteine (SAH) hydrolase 2 | 1.55 | 0.63 |
| AT5G53970 | TAT7, tyrosine aminotransferase 7 | 1.47 | 0.56 |
| AT5G43850 | ARD4, RmlC-like cupins superfamily protein | 1.45 | 0.53 |
| AT4G19020 | CMT2, chromomethylase 2 | 1.40 | 0.49 |
| AT5G14060 | AK2, aspartate kinase 2 | 1.40 | 0.49 |
| AT5G28020 | CYSD2, cysteine synthase D2 | 1.38 | 0.46 |
| AT2G36880 | METK3, methionine adenosyltransferase 3 | 1.37 | 0.45 |
| AT5G17920 | MS1, methionine synthesis 1 | 1.33 | 0.41 |
| AT1G31230 | AKHSDH1, aspartate kinase-homoserine dehydrogenase 1 | 1.32 | 0.40 |
| AT3G01120 | MTO1, methionine overaccumulation 1 | 1.32 | 0.40 |
| AT5G09660 | PMDH2, peroxisomal NAD-malate dehydrogenase 2 | 1.30 | 0.38 |
| AT1G02500 | SAM1, S-adenosylmethionine synthetase 1 | 1.29 | 0.37 |
| AT4G14710 | ARD2, acireductone dioxygenase 2 | 1.23 | 0.30 |
| AT5G43330 | MDH2, cytosolic-NAD-dependent malate dehydrogenase 2 | 1.22 | 0.29 |
| AT5G49160 | DMT1, methyltransferase 1 | 0.80 | -0.33 |
| AT3G13110 | SAT3, serine acetyltransferase 3 | 0.78 | -0.36 |
| AT1G12010 | 2-oxoglutarate (2OG) and Fe(II)-dependent oxygenase superfamily protein | 0.73 | -0.46 |
| AT5G18930 | SAMDC4, Adenosylmethionine decarboxylase family protein | 0.68 | -0.55 |
| AT1G05010 | ACO4, 1-aminocyclopropane-1-carboxylate oxidase 4 | 0.66 | -0.61 |
| AT4G23600 | CORI3, coronatine induced 3 | 0.64 | -0.64 |
| AT1G64660 | MGL, methionine gamma-lyase | 0.45 | -1.15 |
| AT4G26200 | ACS7, 1-aminocyclopropane-1-carboxylate synthase 7 | 0.45 | -1.16 |
| AT2G19590 | ACO1, 1-aminocyclopropane-1-carboxylate oxidase 1 | 0.28 | -1.86 |
| AT2G24850 | TAT3, tyrosine aminotransferase 3 | 0.23 | -2.14 |
